# Neglected Brain Disease—Suicide

**Published:** 1857-07-01

**Authors:** 


### THE JOURNAL

OF

### PSYCHOLOGICAL MEDICINE

AND

### MENTAL PATHOLOGY.

JULY 1, 1857.

### Part First.

### Original Communications.

### Art. I.—NEGLECTED BRAIN DISEASE—SUICIDE.

(BY THE EDITOR.)

It is a question entitled to the serious consideration of the practical physician and medical pathologist, whether there has not been of late years a marked increase in the number of cases of disease of the brain and nervous system? We think the fact is indisputable. Physicians who have favourable opportunities of investigating this subject not only agree in opinion that such diseases are of more frequent occurrence, but that a certain unfavourable (but in its incipient stage, certainly not incurable) type of cerebral disorganization develops itself in the present age at a much earlier period than formerly. Softening of the brain, for example, now often manifests itself at the early age of thirty and thirty-five! It is indeed lamentable that the brain and mind should yield to the influence of certain noxious moral and physical agents, at a time of life when the intellect ought to be in an active and vigorous condition of exercise and health.

We cannot, in this essay, enter at length into an analysis of the causes of so sad a state of cerebral degeneracy. That the brain in the present day is overworked—that its psychical functions are unduly exercised, strained, and taxed in the great effort required, in the severe struggle and battle of life, to obtain intellectual supremacy, professional emolument and status—is a fact which the physician specially engaged in the treatment of this class of maladies cannot ignore. It is difficult to say why that portion of the delicate nervous tissue so intimately and mysteriously associated with the phenomena of mind should be more amenable, in the present epoch, to the influence of those causes which are known to exercise an injurious influence upon the organ of thought. Has the brain deteriorated in its structure? has it less power of resisting the effects of agents brought to bear upon its functions? It is an admitted fact that the type of nearly all classes of disease has, within the last fifteen or twenty years, undergone a material modification. We rarely witness the *acute* and sthenic diseases of our early days, requiring for their successful treatment active and anti-phlogistic remedies. Something is certainly due to the advance made of late years in the science of pathology and therapeutics; but this does not altogether explain the fact referred to. Although the average duration of life *appears* to be greater than formerly, there can be no doubt that the *power of vital resistance has sensibly diminished, and that not only the brain, but other important organs, more readily yield to the influence of disease* The altered habits of society are to some extent dependent upon this condition of the vital organism. However disposed we may be in the present day to exercise a rigid temperance in all that concerns life, the human constitution cannot bear with impunity and safety a great amount of stimuli and mental work. This was not the case in those halcyon days, as they were termed, when men were recognised as being two, three, four, and five-bottle men. This happy change in the social habits of society is certainly, in a great measure, attributable to the social advance of the age and the improved state of morals; but to some extent may not these altered and temperate habits arise from a consciousness of our inability to live above par, as men were accustomed to do thirty or forty years back? We think, in a degree, the fact admits of this explanation. It is, however, the purport of this paper more especially to direct professional attention to the inexcusable neglect with which the affections of the brain are generally treated by the public, and the lamentable amount of ignorance that unhappily exists in the non-professional mind respecting these disorders. This neglect and ignorance is fraught with much irremediable mischief—alas! often leading to the sacrifice of valuable human life. The poor overwrought brain meets with but little attention and consideration when in a state of incipient disorder. The faintest scintillation of mischief progressing in the *lungs*, *heart*, *liver*, and *stomach* immediately awakens alarm, and medical advice and treatment are eagerly sought; but serious well-marked symptoms of brain disorder are often entirely overlooked and neglected; such affections frequently being permitted to exist for months without causing the faintest shadow of uneasiness or apprehension in the mind of the patient or his friends. Morbid alterations of temper—depression of spirits, amounting sometimes to melancholia—headache—severe giddiness—inaptitude for business—loss of memory—confusion of mind—defective power of mental concentration—the feeling of brain lassitude and fatigue—excessive *ennui*—a longing for death—a want of interest in pursuits that formerly were a source of gratification and pleasure—restlessness by day and sleeplessness by night—all obvious indications of an unhealthy state of the functions of the brain and nervous system, rarely, if ever, attract attention until the unhappy invalid, becoming unequivocally deranged, commits an overt act of insanity. Then the exclamation is, "Poor fellow, his mind has been affected for months!" and no one expresses any surprise that he should, in such a state of mental disorder, have hung himself or cut his throat! It is difficult to induce the public to take a common-sense and right view of this important subject; for if the saving of life is our object, it is to the *public* mind we must plainly address ourselves. If a person, in a previous state of mental and bodily health, is conscious that abnormal changes are taking place in the mind—that trifles worry and irritate—that the brain is evidently unfit for work—that the spirits are flagging—that all the evils of life are magnified; if he is disposed to be fanciful—imagining things to exist that have no existence apart from himself—believing that kind friends ill-use and slight him;—if symptoms like these, or analogous to these, are associated with headache, derangement of the stomach and liver, and want of continuous sleep, *the patient may assure himself that the state of the brain is abnormal, and requires careful consideration and treatment.* How often such apparently trifling symptoms of brain disorder precede the fatal act of homicide and suicide! How much may be said for those driven by unrecognised and neglected disorder of the brain and mind to acts of self-destruction!

The sad and premature death of a gifted child of genius, poor HUGH MILLER, has led us into the above train of thought. How mysterious is the act of suicide—how difficult it is to reconcile with our *à priori* knowledge of the instincts of human nature the fact that a person can deliberately commit an act of self-destruction! There is no feeling so strongly implanted in us as the love of life. It is an instinct of nature to strive to preserve our being, and an instinct cannot easily be overpowered and crushed. One of our poets, in alluding to this subject, after declaring life to be the dream of a shadow, "a weak-built isthmus between two eternities so frail that it can neither sustain wind nor wave," yet avows his preference of a few days', nay, a few hours' longer residence upon earth, to all the fame which wealth and honour could bestow:—

"Fain would I see that prodigal who his to-morrow would bestow
For all old Homer's life, e'er since he died till now!"

"Is there anything on earth I can do for you?" said Taylor to Dr. Wolcott, as he lay on his death-bed. The passion for life dictated the answer—"Give me back my youth." These were the last words of the satirical buffoon. There is an anecdote recorded of one of the favourite Marshals of Napoleon, the Duke de Montebello, which finely illustrates the strength of this instinctive principle. During a battle in the south of Germany the Duke was struck by a cannon-ball, and so severely wounded that there was no hope of a respite. Summoning the surgeon, he ordered his wounds to be dressed, and when help was declared unavailing the dying officer, excited into a frenzy by the love of life, burned with vindictive anger against the medical attendant, threatening the heaviest penalties if his art should bring no relief. The dying Marshal demanded that Napoleon should be sent for as one who had power to save; whose words could stop the effusion of blood from the wound, and awe nature itself into submission. Napoleon arrived just in time to witness the last fearful struggle of expiring nature, and to hear his favourite Marshal vociferate, as the lamp of life was just being extinguished, "Save me, Napoleon!" We have heard of a similar instance in humble life. A man on the point of death vowed he would not die, cursing his physician, who announced the near termination of his life, and insisting that he would live in defiance of the laws of nature! In both these cases we see clearly manifested the passion for life, the instinct of self-preservation which it is almost impossible to master.

It is recorded of Louis XI. of France, that so desperately did he cling to life when everything warned him to prepare for death, that he, in accordance with the barbarous physiology of that age, had the veins of children opened and greedily drank their blood; hoping in this way to fan the dying embers into a flame.

So much for the normal, the healthy, and natural instinct, LOVE OF LIFE. Let us consider this instinct in a disordered or perverted state.

The life of the celebrated author of the "Testimony of the Rocks," "The Old Red Sandstone," "My School and Schoolmaster," admits of a psychical as well as a medical consideration. An American contemporary* has so ably analysed the psychological features of this remarkable man, that we find we cannot do better than quote some passages from the sketch referred to. In speaking of Mr. H. Miller's family the writer remarks:—

"In the parentage of Hugh Miller we seem to find support for the popular notion so often referred to, but which statistics have failed to sustain, of the usual preponderance of the maternal stock in the cha-

* "American Journal of Insanity" for April, 1857.

racter of the son. The prominent characteristics of the two maternal uncles appear to have been combined in the nephew; the retentive memory, the love of romance and poetry of the one, with the large reflective faculty and deep religious feeling of the other. From the father, a 'singularly robust and active man,' massively simple, yet not wanting in sagacity, through a line all sea-faring men since the Danish invasion, came the courage, hardihood, and, in part, the superstition of a Norse ancestry. The father was a giant, even among his brethren, and from him was transmitted that strength of physical organization which permitted in the son close mental application, at the same time with the most severe and exhausting bodily labour, throughout a period of fifteen years."

Hugh Miller's singular mental idiosyncracy at the early age of *five* can easily be devined from the following extract from his autobiography. In alluding to the "apparitions of the buccaneer ancestor, and of the dissevered hand," he observes :—

"The fatal tempest, as it had prevailed chiefly on the eastern coasts of England and the south of Scotland, was represented in the north by but a few bleak, sullen days, in which, with little wind, a heavy ground-swell came rolling in coastwards from the east, and sent up its surf high against the precipices of the Northern Sutor. There were no forebodings in the master's dwelling; for his Peterhead letter—a brief but hopeful missive—had been just received; and my mother was sitting, on the evening after, beside the household fire, plying the cheerful needle, when the house-door, which had been left unfastened, fell open, and I was dispatched from her side to shut it. What follows must simply be regarded as the recollection, though a very vivid one, of a boy who had completed his fifth year only a month before. Day had not wholly disappeared, but it was fast posting on to night, and a gray haze spread a neutral tint of dimness over every more distant object, but left the nearer ones comparatively distinct, when I saw at the open door, within less than a yard of my breast, as plainly as I ever saw anything, a dissevered hand and arm stretched toward me. Hand and arm were apparently those of a female; they bore a livid and sodden appearance; and directly fronting me, where the body ought to have been, there was only blank, transparent space, through which I could see the dim forms of the objects beyond. I was fearfully startled, and ran shrieking to my mother, telling what I had seen; and the house-girl, whom she next sent to shut the door, apparently affected by my terror, also returned frightened, and said that she too had seen the woman's hand; which, however, did not seem to be the case. And finally, my mother going to the door, saw nothing, though she appeared much impressed by the extremeness of my terror and the minuteness of my description. I communicate the story as it lies fixed in my memory, without attempting to explain it. The supposed apparition may have been merely a momentary affection of the eye, of the nature described by Sir Walter Scott in his 'Demonology,' and Sir David Brewster in his 'Natural Magic.' But if so, the affection was one of which I experienced no after-return; and its coincidence, in the case, with the probable time of my father's death, seems at least curious."

The American critic considers that

"There, no doubt, belongs to this apparition an important meaning, which its reference simply to a momentary affection of the eye does not suggest. It is well known how largely the supernatural element enters into the philosophy of a rude, and especially a sea-faring people. The legends of the mermaid of the Dropping Cave, and the water-wraith of the Conon, with the hints of witches, ghosts, and 'gude folk,' contained in the book, go to prove that the Cromarty villagers were no exception to the general statement. When we think of the lonely, half-orphan child, precocious in memory, and in the dawning of an imagination which was to become a distinguishing feature of the man, and at last, under the stimulus of deranged function, fatally to prevail over a strong instinct and a high moral sense, such a morbid embodiment of fancy will not excite our wonder. In the case of a child the phenomenon would most likely be of the nature of an illusion, and as such might, perhaps, have been easily explained. The night, the loneliness of the situation, the atmospheric conditions attending the tempest, and the roar of the waves as they smote against the cliffs, were sufficient exciting causes of the apparition. We shall see that the peculiar mental disposition grew with the advancing years of the lad.

"The subject of hallucinations is one that cannot attract too great attention, and within the past few years there has been much written upon it that is interesting and valuable. That visual hallucinations may occur under a normal condition of the perceptive and judging faculties, and hence are consistent with reason, is readily admitted. Not so, however, the proposition maintained by high authorities, that a large class of these phenomena are purely physiological. We must consider that all hallucinations in which the phantasy has an objectivity—and those instances of prolonged visual impressions of which we are conscious on closing the eyes, after gazing intently upon a bright object, have not this character—all true hallucinations are symptomatic of deranged function or of organic disease, and are truly pathological in their nature. The phantoms of the child, born with a diseased organization, or reared in an atmosphere of superstition—the dreams of the disturbed sleeper—the ecstatic creations of lovers, poets, and illuminati—the fearful impressions of mania and delirium—the embodyings of rage, fear, and remorse—incubi from a disordered viscus, and muscæ volitantes from simple retinal disturbance, are essentially morbid manifestations. Against this view, which seems necessary to the practical treatment of the subject, it is mainly urged that the hallucinations of certain great men are inseparably connected with the important moral and scientific truths which they have developed. But a minute and complete history of the man generally affords, as in the unhappy instance under notice, the clearest evidences of their morbid nature."

The mind of Hugh Miller exhibited early symptoms somewhat allied to mental aberration. He says in his autobiography, when referring to the effects of the first few months of his apprenticeship upon his mental condition :—

"Though now seventeen, I was still seven inches short of my ultimate stature ; and my frame, cast more at the time in the mould of my mother than in that of the robust sailor, whose 'back,' according to the description of one of his comrades, 'no one had ever put to the ground,' was slim and loosely knit ; and I used to suffer much from wandering pains in the joints, and an oppressive feeling about the chest, as if crushed by some great weight. I became subject, too, to frequent fits of extreme depression of spirits, which took almost the form of a walking-sleep—results, I believe, of excessive fatigue—and during which my absence of mind was so extreme, that I lacked the ability of protecting myself against accident, in cases the most simple and ordinary. Besides other injuries, I lost at different times during the first few months of my apprenticeship, when in these fits of partial somnambulism, no fewer than seven of my finger-nails. But as I gathered strength my spirits became more equable ; and not until many years after, when my health failed for a time under over-exertion of another kind, had I any renewed experience of the fits of walking-sleep."

Symptoms of severe bodily fatigue, associated with extreme depression of spirits, mental exhaustion, reverie, paroxysms of melancholy, partial somnambulism, to say nothing of the hallucinations manifesting themselves at so early a period of life, should have been viewed as important *psychical deviations from health requiring the most careful and cautious moral and intellectual training, medical and hygienic treatment.* As he advanced in his apprenticeship he became "desponding, apprehensive of an early death, and his gloomy temperament, with the superstition of his nature and education, laid powerful hold of his mind."

Hugh Miller says in his autobiography :—

"One day, when on the top of a tall building, part of which we were throwing down to supply us with materials for our work, I raised up a broad slab of red micaceous sandstone, thin as a roofing slate, and exceedingly fragile, and, holding it out at arm's length, dropped it over the wall. I had been worse than usual all that morning, and much depressed ; and, ere the slab parted from my hand, I said—looking forward to but a few months of life—I shall break up like that sandstone slab, and perish as little known. But the sandstone slab did not break up ; a sudden breeze blew it aslant as it fell ; it cleared the rough heap of stones below, where I had anticipated it would have been shivered to fragments ; and, lighting on its edge, stuck upright, like a miniature obelisk, in the soft greensward beyond. None of the philosophies or the logics would have sanctioned the inference which I immediately drew ; but that curious chapter in the history of human belief which treats of signs and omens abounds in such postulates and such conclusions. I at once inferred that recovery awaited me; I was 'to live and not die,' and felt lighter, during the few weeks I afterwards toiled at this place, under the cheering influence of the conviction."

We cannot resist quoting an important passage *in extenso* having reference to the psychological bearings of the case of Hugh Miller:—

"Most efficient in producing morbid mental phenomena, next to bodily disorder engendered by reckless inattention to the organic functions, is the exercise of abstract thought; of which an old writer says, that it 'dries the brain and extinguishes natural heat.' For the latter half of the second period of Hugh Miller's education, when his bodily health had become robust, and when his mental labour was little more than the reproduction of his reading and observation, we have no account of hallucinations. When, however, the character of his writings passes from that of descriptive and legendary tales to the generalizations of his later geological works, we find a continually enlarging development in the direction of his peculiar infirmity. That the abstraction of genius, so prolific of the grandest truths, should have so much in common with the reverie of mental exhaustion and depravation, is a curious and humiliating fact.

"Having thus far referred, in these remarks suggested by the suicide of Hugh Miller, to what will probably be thought its true explanation, in the peculiar mental constitution and early history of that great man, we have nearly done. The growing manifestations of mental disturbance which followed the total neglect of regimen, exercise, and sleep, and his unintermitting abstraction and deep thought, were such as characterized his former experience. They took the form of suspicions and apprehensions, which, at first connected with enemies from without, and with hallucinations of the senses, grew rapidly more intense as the inducing causes were continued, until they came in paroxysms of maniacal terror with the vague and dreadful visions of nightmare. There were also the somnambulism and the sense of extreme exhaustion which attended the attacks of his youth. But we will not dwell upon the particulars of the fatal termination. With these our readers are already acquainted. The immediate history of the event, connected with one of so clear an intellect, so conservative religious belief, and such unstained character, has been studied with a mournful and rational interest. If such an one be not safe from a calamity so terrible, how great reason to fear have those of a feebler intellect, of stronger passions, of vacillating belief, and unfortunate lives! It is in behalf of this interest that we have traced, beyond the facts which give rise to these suggestions, the causes of the suicide in the mental organization of the man. Let us proceed from this view to notice particularly the character of the act.

"Psychological science at the present day, returning from the material tendency which it had acquired through the German philosophies, refers largely to moral causes in its interpretation of suicide. In no other way can its history, as presented under the civilizations before the Christian era, and since among the Asiatic communities, be made consistent with its evident present relations to science and religion. Nothing can be clearer than that the great mass of suicides noticed in ancient history were connected with the philosophies and false religion of their time; and it can hardly be doubted, that as these had their birth in a moral darkness, so, to a fearful extent, do those of our own age have their source in a moral depravation.

"Yet, the common sense of mankind in all ages has recognised the suicide of disease, and has made the divisions of the suicide of sanity and the suicide of insanity. In the light of modern science we may view the former as rational, and as passionate; the latter as the determined, and the impulsive. The ancient civilizations afford us numerous instances of the rational suicide. Viewed in the imperfect moral light of their age, some of these illustrate the noblest virtues of the human character. This class can scarcely be admitted where the Christian religion, even in its most corrupted forms, has extended. Its inspired teachings have created a moral sense averse to self-murder, as universal as the instinct of self-preservation, and as powerful as that which forbids homicide. A depraved morality is now, without question, the most prolific source of suicide in the civilized world. Ours is the age of the passions, and the question of suicide of this class has become of alarming magnitude and importance. But this is the province of the moralist and the divine.

"What we have called the determined suicide of the insane is connected with chronic mental disease, usually lypemania, and the disposition to it is a symptom which claims for its unfortunate subject the most special and discriminating treatment. Some rare and not well-understood cases have led to the admission of a suicidal monomania. But it is in maniacal suicide, and its connexion with functional derangement and structural disease, that we find matter of especial interest and importance. It is of the deepest interest, because the subjects of the fatal disposition are so often those whose genius and virtues claim the admiration and homage of their fellow-men,—of the first importance, because the errors that induce the transient insanity which belongs to it tend, in a thousand other directions, to death, and because it seems so possible in a great degree to effect their prevention.

"There has been no hesitation, on the part of the public, as to the nature of the act by which Hugh Miller has been lost to the world. That it was done under the most overpowering and terrible maniacal delusions, perhaps no one has doubted. A general knowledge of the man and the circumstances of the fatality have sufficed already for this conclusion. What we have found in the history of an early-developed idiosyncrasy and its prominence through a long period, in his education, in his changed mental and bodily habits, and in the symptoms which preceded the final paroxysm, may perhaps serve to strengthen the grounds of that judgment. We shall at least be satisfied from the fact that any inquiry into such a subject must aid to enforce the important moral with which it is charged. Here was one in whom no morbid cravings for the unknowable swayed a humble belief in the sufficiency of revealed truth to meet the moral wants of our race, while his noble intellect permitted the largest conceptions of Deity in his works as Creator and Governor of the universe. And still, one who, while extending the science of the world's creation thousands of years into the past, and rearing it in bulwarks about the infinitely important domain of his religious belief, yet left unstudied the immediate and underlying truths of his own mental organization, and that complex and delicate machinery, through which the spiritual essence must elaborate all that we can know of life, to derangement and a terrible dissolution."

So much for the unhappy suicide of this valuable and gifted member of society. But is this an isolated case? In looking back upon the past few years can we not recall to mind many similar and sad illustrations of suicide, clearly consequent upon a non-recognition of the important fact that, *in every case of departure from a normal or healthy action of the mental and moral powers, the brain is in a physical state of derangement* —that disturbed thoughts, depression of spirits, delusive ideas, hallucination of the senses, alienation of affection, exaggerated fears and apprehensions, morbid impulses, *have the same relation to the brain that disordered respiration has to the lungs*—and that it is as impossible for the faculties of the mind to be disordered or deranged independently of the brain, *as for the respiration to be affected apart altogether from a change in the material condition of the lungs and heart?* It is well observed by an able writer experienced in the treatment of insanity (the late Dr. Mann Burrows):—

"Did mental derangement experience the same prompt attention as most other complaints, it is impossible to judge how much more favourable the results might prove: the reverse almost always obtains; and therefore insanity more frequently degenerates into a chronic and continuous type before remedies are applied. All practitioners have remarked how difficult it is of cure where it has taken the latter form comparatively with acute or recent cases. In this disposition it but assimilates to other diseases. For be the disease what it may, whenever remedies are neglected after its first access there is great danger of its assuming an obstinate, if not a permanent character. Unfortunately the approach of insanity, though generally perceptible to strangers, is rarely remarked by relatives. We are all apt to shun that which is painful or displeasing; so the insidious approaches of mental derangement are rather construed into nervous irritability, or eccentricity, or anything rather than the truth, and are suffered to proceed till some terrible exacerbation of delirious fury or despondency ensues. A malady is thus often confirmed in one whom we most value, and whose intellects very probably might have been preserved had timely aid been administered. How frequently do we witness the bitterness of self-accusation, and the unceasing regrets of the near connexions of lunatics, because they have persevered in their wilful blindness till the calamity they deprecated has occurred! Assuredly the approach of intellectual disorders sometimes escapes the most intelligent observer; while bodily ailments, from the derangement of some ordinary function or from acknowledged pain, are at once visible."

We have, with a view of establishing beyond the possibility of doubt the important fact, that an enormous sacrifice of valuable human life annually takes place in this and other countries owing to culpable and inexcusable negligence and ignorance, collected from the usual channels of daily intelligence a number of cases of suicide, in *all of which there existed before the act of self-destruction well-marked symptoms of physical ill-health and disorder of the brain and nervous system.* In every one of these cases, no suspicion appears to have been entertained as to the *mental health* of the unhappy suicide until life was extinct! Had the *lungs, heart, liver, stomach,* or *skin* exhibited equivalent symptoms of a departure from health, would not medical aid have been, without scruple or loss of time, obtained? Alas for the poor brain! Its functions, its psychical or mental manifestations, may be palpably deranged, and yet awaken no apprehension on the part of those immediately in association with the invalid. This subject is of such vast importance when viewed in connexion with the SAFETY OF HUMAN LIFE, that we do not hesitate in placing before our readers, even at the risk of repeating a thrice-told tale, a body of valuable evidence clearly and conclusively illustrative of the fatal consequences resulting from the neglect of *positive, well-marked, and obvious symptoms of brain and mind disorder.* We deliberately and unhesitatingly assert, having had no inconsiderable experience in the treatment of mental derangement associated with a tendency to suicide, that nearly all these fatal instances of self-destruction might have been averted if the patient had been brought within the reach of remedial measures; in other words, *if the abnormal or unhealthy state of the brain, as indicated by a disturbed state of the thoughts, mental depression, &c., had been fully recognised and properly treated by medical and moral agents.* How difficult, however, it is to persuade those ignorant of the physiology and pathology of the brain and mind, of the necessity and importance of attending, without delay, to the earliest scintillations of brain mischief and disorder! It is at this period when so much may be effected for the relief and probably for the positive cure of the patient. But we proceed without further comment to the publication of some sad illustrations of neglected brain disease leading to suicide and death. These facts will, we trust, speak trumpet-tongued to all interested in the treatment of diseases of the brain and in the important subject of the preservation of human life.*

Colonel C—— B——, aged 55 years, of the East India Company's service, committed suicide by cutting his left arm with a razor. One morning deceased rang the bell, and on the housemaid answering it, he requested her to take a note to Colonel P——. She returned in about twenty minutes; she asked the ladies where the colonel was. They told her he was in the parlour. On going there she could not find him; she then went to the back drawing-room, and knocked at the door, but received no answer. Colonel P—— arrived, and having made known her suspicions to him, he had a ladder brought and entered at the window. Opened the door for witness. Upon entering she found her master sitting in his chair covered with blood. Two pistols were on the floor, and a razor covered with blood was on the chair at his side. His left arm was cut, and he seemed quite dead. *Deceased had been very low-spirited* for two or three days. Colonel P—— said *deceased had been greatly depressed lately*. He received a letter from him the day before his death, in which he said, "*I don't think I shall ever know happiness again.*" Believed his depression arose from his being separated from his wife.—Verdict—Temporary insanity.

C—— H——, 41 years of age, steward of a steam-packet, destroyed himself under the following circumstances. The deceased had been in the steam-packet service for ten years, and returned home from his voyage in *a very depressed and melancholy state*, but appeared to recover towards night. The following morning the deceased showed no inclination to get up, and his wife urged him to do so, and occupy himself with his duties on board, by which means she thought he might recover from his depression of mind. He told her in a very melancholy tone he could not do so, and gave her directions to forward some things which she had prepared for the passengers' use by one of the neighbours, with a message to the captain that he had become insane. Finding all her persuasion useless, she did as she was directed; but had scarcely entered the sitting-room, when she was horrified at seeing her husband come out of the bed-room with a clasp-knife in his hand, and the blood flowing profusely from his throat, which was cut across to a depth of several inches; and, after articulating with difficulty, "Well, old girl, I have done it perfectly now," he staggered forward a few feet and fell upon the sofa. After lingering a few hours, during which he was speechless, he expired. He had always treated his wife and two children with the greatest affection; *but he had for a long time past so repeatedly expressed his intention to destroy himself, and exhibited such confirmed melancholy and thoughtfulness, frequently sitting for*

---

*Cases of suicide occasionally occur without any apparent precursory symptom of actual brain or mental disease, but these cases are exceptional only. The subjoined is one in point. Mr. George Allen, clerk in a store in New Philadelphia, Ohio, recently committed suicide. He was a young man of good habits, moral, honest, much respected, and bid fair to become a useful member of society. The subjoined account of his conduct exhibits a sad instance of the mental and moral perversion and infidel apathy now but too current. "The day before he committed this rash act, he appeared to be in good spirits, called on some of his friends and told them he intended to go home, paid all the debts he owed, returned the books he borrowed, and all the time talked and laughed with his accustomed gaiety. In the evening previous to his death he wrote several letters, and composed the epitaph he wanted on his tombstone. He attended to the customers in the store, and no one could judge from his conduct the silent determination within. In the course of conversation with some young gentlemen, one of them asked 'What is life?' Deceased good-humouredly replied, 'he intended shortly to solve that problem.' It was evidently his intention to put an end to his existence by taking chloroform. He purchased a bottle of it; made his bed on the counter, lay down, took an overdose, but it had not the fatal effect. He then got a ladder and rope, and hung himself in the loft of the new building adjoining the store. His toes touched the floor; his hands were untied; he swung aside of the ladder, so that he probably could have saved himself even after he had taken the fatal leap."—*From a recent American Paper.*

*hours in fits of abstraction without uttering a word, that she had been obliged upon several occasions to walk about the streets all night for fear that he might murder her.* It appears that his mother had about two years previously unexpectedly destroyed herself in a shocking manner. This made a deep impression upon the mind of the deceased, and induced a morbid feeling, which caused him to think that two other persons in the company's service were endeavouring to procure his discharge for their own advantage (delusion). There was no foundation for this impression.—Verdict—Temporary insanity.

P—— (an old soldier) had been remarkable for his happy and kindly disposition. Three weeks previously to his death his niece observed that *he was unusually depressed in spirits,* which so increased, that on Sunday se'nnight Mr. J—— P——, surgeon, was summoned to attend him. That gentleman found him suffering from great determination of blood to the head. A few mornings afterwards he rose about seven o'clock, and went out of the house, and in less than half an hour he was found in the hayloft, his head being held by a cord tied to a beam five feet from the ground, and his body in a reclining posture. Death had not, however, resulted from hanging; deceased's hands were bloody; and it would seem that finding his attempts at self-destruction in that way were not effectual, he inflicted so deep a wound in the throat with some sharp instrument as to sever the windpipe and all the large bloodvessels of the throat. The servant girl, to whom he spoke on the way out, seems to have had no suspicion of his intention.—Verdict—Temporary insanity.

Mr. R—— R—— K—— committed self-destruction by cutting his throat. It appeared upon inquiry that the deceased had for some time past been labouring under delusions that some persons owed him a large sum of money; and recently, on getting ready to go to church, he suddenly turned round and asked those who were standing near to him, "What, do you mean to assassinate?" *He was constantly entreating his friends that he should not be sent to a lunatic asylum, although nothing was ever said to him on the subject.** On the Thursday night he was out with his aunt, and they returned home together. *In consequence of his low spirits, his aunt had sat up for some nights with him until he was asleep; but on the Thursday night, at his urgent request not to leave him, for fear, as he said, that some one would take him away,* she remained with him all night, until eight o'clock in the morning. She then left the room for a few minutes, and on her return she found that he had taken advantage of her temporary absence, having in that short interval cut his throat with a razor. All the vessels of the neck were divided. He was a very steady young man; and although his friends had often tried to find out the cause of his melancholy, they had never been able to do so.—Verdict—Temporary insanity.

J—— N—— committed suicide by drowning himself in a stream near Windsor. The deceased was 28 years of age, and had gone about his work as usual on the morning of the day above mentioned, and when he left home proceeded in the direction of Virginia Water. At noon he was observed by one of the park-keepers to throw himself off the high bridge near the Blacknest entrance to the Royal property. Deceased had written on the wall, "Good-bye all. J. N." No cause could be assigned for the rash act; *but the melancholy annals of the deceased's family show a remarkable and almost unaccountable predisposition to self-destruction on the part of its members.* About twelve years ago a brother of the deceased threw himself from off the same bridge, and was drowned. Twelve months previous to the death of the deceased, a cousin drowned himself in the same water; many years ago an uncle hung himself in an adjoining wood; and about seven years ago another cousin hung himself in a plantation on the Silwood estate, close by.—Verdict—Insanity.

A—— B——, aged 17, committed suicide by drowning. *She had been in low spirits for some weeks. She was labouring under a nervous complaint.* On Sunday evening, the 28th, she went to bed at seven o'clock. One of the witnesses found her sitting up in bed and holding her forehead in her hand. *She said she had a*

* How often does this consciousness of insanity exist! the unhappy patient fully recognising the importance of being placed under restraint.

*very bad headache. She did not sleep the whole of the night, and behaved in a very strange way.* The next day, the 29th, between twelve and one o'clock, she attempted to get up, and the witness persuaded her to return to bed, which she did, and remained till five o'clock, when she got up, complaining of a pain in her bowels, and said she must go out. She complained of the cold, and put on all her clothes, and went away, saying she should not be gone long; she did not, however, return, and witness did not see her alive afterwards. Search was made after her, but she was nowhere to be found. Witness could not account for her lowness of spirits, and thought it was partly constitutional. She was always weak and delicate.

Another witness spoke as to seeing her come from her home about twenty minutes before six in the morning, over a bank, and go down the road leading to the pond in which her body was found.

Another witness, the widow of a deceased uncle, stated that in the beginning of April last deceased and herself were passing a pond called "Little Burnett's pond," when deceased said, "Look here, Hannah; this would be a good place for some one to jump in, and some of these days I shall come and jump in." Witness told her not to have any such thoughts. Deceased had several times complained to her of her head. On the Sunday deceased told her her head did not ache, but felt muddled. The brother of the deceased deposed to pulling the body out of the water with a hay-rake; she was quite dead.—Verdict—Temporary insanity.

On the 7th of June, 1854, an inquest was held on the body of H—— P——, who committed suicide by drowning herself on the 5th of June, at Chipping Sudbury, Gloucestershire, in a pool of water. It appeared that the deceased was last seen alive on the evening of the 5th instant, about ten o'clock, and nothing was heard of her until the following morning, when she was found lying in the above pool. She appeared, when taken out of the water, to have been dead several hours. *Her conduct had of late been strange and eccentric.*—Verdict—Temporary insanity.

Sir J—— M—— committed suicide by shooting himself with a pistol. It appeared that the deceased gentleman's bodily health had of late been tolerably good, *but that his spirits had for some time past been very much depressed.* On Sunday, the 17th, he appeared somewhat better, and retired to rest between eleven and twelve o'clock. Shortly before five o'clock the following morning his valet was awakened by a deadened report of fire-arms, and upon going to his master's chamber he discovered him lying prostrate on the carpet, weltering in his blood, and lifeless; his head was shattered to atoms, and a small double-barrelled holster pistol, which had recently been discharged, was lying close to his right hand.

G—— S—— D——, aged 35, committed suicide by discharging a pistol through his head, under the following determined circumstances.

Mr. H. Prendergast, the barrister, said that he had known the deceased many years, and that he was for some years at M—— and G——, employed by the Government as civil engineer, which profession he had relinquished about twelve months since. In September witness had a communication with deceased and his family with reference to his pecuniary affairs, as he was in a state of considerable embarrassment. He had raised some money on a reversionary interest which was nearly expended. These difficulties had *weighed upon his mind, and he had lately been labouring under great despondency.*—Verdict—Temporary insanity.

F—— J—— B——, a very respectable tradesman, 40 years of age, committed suicide by shooting himself through the head. His two sons, his only children, had emigrated to America. From the period of their departure *his mind became very depressed, so much so as to excite the apprehension of his friends.* One morning he arose at his usual hour and went about his business, giving directions to his workmen. Between nine and ten o'clock, not having come to his breakfast, search was made for him, when it was discovered that one of the upper workshops was fastened, and being forced open, the body of Mr. B—— was found lying upon the floor quite dead—the greater portion of his skull and brains being blown about the room, and the gun lying with the barrel on a vice, and some string fastened to the left hand of the deceased.

Mr. W—— L—— M—— L—— shot himself with a pistol. He had been at least a whole year in a very desponding state, which arose from his thinking he had lost all his property. *He had some months previously attempted to commit suicide; he was sane on every point except money matters.* The brother of the deceased said that the deceased had been suffering mentally for more than twelve months. The disorder commenced some time after a mill (that formed a principal part of his business) was destroyed by fire, and he had an erroneous impression that he was in consequence going to ruin. He had dined at witness's house the previous day, but he tried to avoid any other company. "*My impression is that his mind was decidedly wrong.* I was never informed of what took place in March last until some considerable time afterwards as to his having attempted suicide, when he told me himself, and extracted from me a promise that I would never mention it to any other person." A paragraph from a letter to the witness from the deceased was read by the coroner, as follows:—"I have written this letter with great difficulty, as you may suppose, when I have the awful scene before me. Your affectionate brother, &c." The coroner said that the deceased alluded in the letter to the circumstance that he was watched. The witness said that he was obliged to be looked after, and that his candid opinion was that the business was too great for him to carry on. His affairs were not in the least deranged, but he fancied that the figures were conjured up to deceive him.—Verdict—Temporary insanity.

J——C——S——, ale-merchant, committed suicide by shooting himself through the head with a gun. The deceased was 38 years of age, and the cause of this distressing case of self-destruction is said to have been domestic affliction of a most painful nature, affecting his domestic peace.

It appeared from the evidence that the deceased gentleman *was under such heavy depression of spirits as to cause the greatest uneasiness to his friends and relatives, from the cause above stated.* He had been for some time on a visit with his family, and on Tuesday, rather unexpectedly, returned to N—— B——, when he complained of feeling very low. About five o'clock in the evening he went into the brewery, and had some conversation on business with his brewer, Henry Shaw, after which he went upstairs into his office, and a few minutes afterwards a report of fire-arms was heard. Shaw immediately hastened to the office, and to his great horror found his master sitting in a chair quite dead; the discharged gun, which he had placed to his head, resting against a brick arch, and a small poker which he had applied to the trigger lying across his lap. The face and head were fearfully mutilated, and not a sign of life remained.—Verdict—Temporary insanity.

W—— W——, aged 33 years, committed suicide by hanging himself in his cell. The deceased had been tried at the sessions previous on two charges, of stealing an order for 1400*l.*, and a piece of paper of the value of one penny, and was sentenced at the following May sessions of the Central Criminal Court. Judgment having been respited upon a point of law, he was brought up for judgment at the July sessions, and sentenced to be transported for ten years.

Dr. McMurdo, the surgeon of the prison, stated that when the deceased was committed he was bordering on a state of *delirium tremens. He had been a great drinker. He was continually complaining of headache, and was very excitable.* —Verdict—Temporary insanity.

J—— S—— committed suicide by hanging himself. He went into the cow-house about half-past twelve to clean it out, and was discovered hanging from a beam by a rope. When cut down he was found quite dead. *He had complained lately of suffering in his head.*

A witness deposed that deceased had been in a very desponding state of late. He complained of a pain in his head, and of the times being so bad.

The surgeon stated that the deceased since November last was suffering under *great prostration of strength and want of sleep, and complained of disturbing thoughts when he was awake, crying like a child, and in very low spirits.*—Verdict —Temporary insanity.

Miss M—— F——, aged 35, a singer and teacher of music, committed suicide by hanging herself by a cord fastened to the rails of the stairs. *She had been in a desponding state for three months,* the origin of which was attributed to a supposed disappointment in marriage; but it appeared from the evidence that the gentleman was no more than a spectre bridegroom. Her father was at the time in a lunatic asylum.

Hannah Gothart, of Claypit-lane, and who lived next door to Mrs. F——, said, "My child came into the house on Monday afternoon at four o'clock, and said that Mrs. F—— was in a fit. I heard her screaming at the same time. I went to the house, and Mrs. F—— cried out, 'Oh, my daughter is hung up!' Two neighbours came in and went upstairs. I went with them, and saw the deceased suspended by a cord fastened to the rails of the stairs. One of the men lifted the body in his arms, and I cut the rope. She was quite dead. It was about four o'clock in the afternoon."

M—— S—— stated as follows:—"Mrs. F—— is cousin to me by marriage. Her husband is not dead. He was a very bad man, and they left him and came to Leeds twenty years ago. I believe he was in a lunatic asylum. I have seen Miss F—— every week for three months, *and thought her very low.* She told me something about a disappointment. She asked me some time since to prepare the wedding breakfast, as she was going to be married, and I did so. It was in June last. The breakfast was prepared, and Miss F—— and her bridesmaid were dressed by eight o'clock in the morning, but no man came. I tried to cheer her up, and asked her to write to the young man, as he might be ill. She said no, they had had no notes, and she would not write. She said he was to bring the carriage with him. She did not mention his name. She said he lived at Headingly. *She was very much distressed, cried bitterly,* and was obliged to go to bed. The mother too was very much distressed. She had been deluded, I believe. A number of dresses had been ordered, and came up to the house. The young man was never seen at the house. She owned that he had never been at the house, but she had seen him at concerts or some of those places. She was a singer or teacher of music, and she met him, I understand, at some musical society. *I have thought her not quite right for three months.* I saw her on Sunday last; *she was very low then,* and said she could not find courage to go out. She said that some one had met her, and told her she had made a sad mess of herself *She was sadly cast down, and appeared to labour under a delirium.* Wine, bridecake, elegant dresses, and other things were prepared. I have no doubt she has not been right in her mind for a long time."

Mrs. Lockwood, another witness, deposed that she had known the deceased fourteen or fifteen years. "For the last three months I don't think she has been altogether right. She had told me she had a disappointment in marriage; that she had taken a house in Chapeltown. I asked her if she had seen it, and she replied in the negative. I asked her who the gentleman was, and she said I should know soon enough; that there had not been any letters; that he had not been at her house; and that it had all arisen from spirits and flowers. I asked her what she meant, and she replied that I could not understand, and got into a rage. I met her on Saturday last. She said she could not get over this great disappointment. I told her I thought it was a delusion, and advised her to think less of it. She began to talk very wildly; twisted her thumb nearly round; and when I asked after her appetite she became very much enraged. *I am satisfied she was out of her mind,* and on Saturday afternoon she looked very wild indeed." —Verdict—Temporary insanity.

W—— C——, aged 40 years, was found dead, and hanging inside a hollow tree, in Hyde Park. In his pocket a paper was found, with the following written in pencil: —"I die a victim, and not a self-murderer;" and "W—— W——, P——, near Botley, Southampton." A letter was received from Mr. W——, to the effect that a man of the deceased's description had been his clerk, but had *left his situation, wandering no one knew where, and that everybody who knew him was not surprised at what he had done,* he latterly having been thought of insane mind. —Verdict—Temporary insanity.

J—— S——, a weaver, aged 68 years, committed suicide by hanging himself during the temporary absence of his wife. Upon the inquest the following evidence was given:—

J—— S——, the wife of the deceased, stated that her husband worked at his trade as a weaver in the Oracle nearly all his life. *Latterly he had not much to do, and was greatly depressed in spirits.* He had until lately enjoyed very good health, and was not attended by any medical man. Yesterday morning he went out about nine o'clock, without taking his breakfast or speaking to me, which was very unusual. He came in just before twelve, and I asked him where he had been. He replied, 'I have been wandering about, but have not been into anybody's house.' He complained of being cold and of his head aching, and said he was afraid he should lose his senses. We then had a bit of dinner, and he still complained of his head. At a little before four o'clock we took tea; he read a little, but again said his head was bad. We went to bed about nine o'clock; he was very restless during the night, got up several times, and complained of a pain in his head. I got up just after seven o'clock, leaving him in bed, and went down stairs to prepare the breakfast. I took him up a cup of tea and some toast, and I observed that his hand trembled very much. I left him in bed and went out about nine o'clock to Mr. Brown's, in Friar-street, to pay my rent; and I said to my husband before I went out that I would lock the door, and he was to lie until I returned. He said, 'Ah, do.' I then went away, and returned just before ten o'clock, unlocked the door, and went up stairs. I spoke to him, but received no answer. I looked up, and saw he was hanging to the bedstead by a little bit of cord. I took a knife out of my pocket, cut the cord, and he fell on the floor. I then went down stairs, and ran over the way to one of the neighbours and gave the alarm, and on my return found he was dead. He was a good man, and a good husband, and we had no previous quarrel. He had been in this low desponding way for eight or nine months, but worse lately. He used to say, when the Oracle was lost his family would starve. He had often expressed himself to the effect that he should go out of his mind; but he would not allow me to go for any medical man, as he said it was his mind."—Verdict—Temporary insanity.

A medical gentleman, Mr. J—— S——, aged 42 years, committed suicide by hanging himself. It appeared from the evidence on the inquest that the deceased had taken apartments at the hotel of a Mrs. Carroll, the Ivy tavern, Bridge-place, Harrow-road, and desired to be called at six o'clock in the morning, in order that he might return by train to Bath. As he did not answer, although the servant man repeatedly called at his door, it was forced open, and he was found suspended by his handkerchief from the bed-post and quite dead. Mr. John Hunter, a friend of the deceased, said that the deceased arrived in London a fortnight since in a very desponding state, frequently exclaiming, "My brain is burning, my brain is softening; I fear my wife and child will be reduced to poverty." Although he thus feared for his family, he was worth 1300*l*. He always expressed a conviction that the softening of his brain would kill him, yet he would not see any medical man. In his portmanteau the following letter to his wife was found:—"I have missed the train again. Oh, God! I know not what to do, what to come home for. My brain is burning and getting softer every day, and I have no senses left. I have often prayed to God to give me my intellects. Oh, what will become of my wife and child! I have no friends left."—Verdict—Insanity.

An inquest was held at Chatham on the body of J—— B——, aged 40, who destroyed herself by hanging.

The surgeon stated that he had been in the habit of attending the deceased for the last year and a half. He saw her on Thursday while she was out walking, when he observed her to be walking very slowly, and appearing to be very much depressed in spirits and very desponding. Witness on that occasion put some questions to her, but she replied that she did not feel ill, but uncomfortable in her mind, and very anxious. She at first said there was no cause for her depression, but she could not shake it off; that the family in the house had noticed the same thing, and had spoken to her about it.—Verdict—Temporary insanity.

J—— W—— B—— committed suicide by hanging himself. At the inquest the widow of the deceased deposed that he was her husband, to whom she had been married about three years. That he usually slept very little at night, and she had wished him to obtain medical assistance, but he replied that a doctor could do him no good, as he had no pain, and he hoped he should soon be better. He looked vacant at times, and occasionally very wild. On Monday afternoon last he went into the dining-room, where witness and her daughter were sitting. He looked at them, but did not say anything. His face was very red, and he appeared very much excited. She inquired of him what was the matter, and endeavoured to persuade him to tell her what troubled him; after some hesitation he said that everything went wrong, and there seemed to be a blight upon everything he did. He had recently been much troubled about his machinery, which had cost him much more money than he had expected. On the previous morning he appeared very restless, and kept going in and out of the rooms, apparently much disturbed, and witness felt certain he was not in his proper mind at the time he hanged himself, and that he had not been so at intervals for several days.—Verdict—Temporary insanity.

N—— M—— committed suicide by suspending himself by a rope. The deceased had lived in the service of Mr. Turner for the very long period of nearly forty years, during the whole of which time he had been considered a man of strict integrity. Recently his employer had been robbed of a large quantity of rags, and as it was the duty of the deceased to take care of his master's property, the loss appears to have affected his spirits, so as to render it necessary to watch him. On Monday morning, about six o'clock, his son found him in a secret place in great distress of mind, and he solicited his son to hang him out of the way. After endeavouring to console him, and telling him to banish such thoughts from his mind, his son went about his business. About two o'clock in the afternoon he was missed from home, and on searching his son found him as before described. At the inquest it appeared that the deceased had been in a desponding state for some time past.—Verdict—Temporary insanity.

J—— H—— P—— committed suicide by hanging himself in his bed-room. At the inquest holden it appeared from the evidence that the deceased had been in a desponding state for a considerable time; that he was occasionally insane; and that insanity was hereditary in his family. This having been satisfactorily proved, the jury returned a verdict of temporary insanity.

S—— P—— aged 60 years, committed suicide. It appeared from the evidence that the deceased had been in a desponding state for some time. The surgeon deposed as follows:—She had at times been very excited, sometimes very depressed, low-spirited, and dejected, and always fretting about something or other. Sometimes she would say she had no clothes to wear, at other times that her children were starving. This had been the case for the last six weeks or two months. I have had three different persons at times to look after her, but they had all gone away, the last one on Saturday last, ill; she had been worse lately, but on Sunday seemed more rational. She was very much excited yesterday (Monday) and irritable, but better at night. Early this morning she was restless and could not sleep, but there was nothing particular in her manner; sometimes she knew what she was about, and sometimes she seemed lost. She dreaded having a stranger to wait upon her.

Another surgeon stated that he had known the deceased, and had personally attended her four or five years or more; that he had attended her once or twice a year when her liver or stomach was out of order. She used to be low at times, and at other times irritable and excited. "I saw her within this fortnight, and found her in a very excited state, and talking in an incoherent way; she looked very vacant. I talked to her sometimes, and left her more tranquil. I considered she was suffering from nervous irritability; she has usually when I have seen her been low and silent, but the last time she was irritable and excited, and said she would not have any one with her. She was worse then than I had ever seen her before. I saw her this morning; she was quite dead. I found the mark of a cord round her neck, no doubt produced by the cord fastened to the bedpost. No doubt her death was caused by strangulation, by the pressure of the cord on the windpipe." —Verdict—That the deceased destroyed herself while in an unsound state of mind.

W—— J—— committed suicide by tying a cravat tightly round his throat, and used part of a two-foot rule to make it tighter, so as to produce strangulation. It appeared that deceased was in a very destitute condition, and complained much of a pain in his head. He was a married man, but his wife had left him. It also further appeared that his father committed suicide by hanging himself, and in the same parish.—Verdict—Temporary insanity.

A person named A—— committed suicide by throwing himself on a railway, the train running over him. The deceased was between 40 and 50 years of age, and, in consequence of reverses in business, had become somewhat reduced. The latter event appeared to have preyed on his mind, so as to impair his intellect. His friends, anxious that he might be properly taken care of, placed him under proper surveillance in the union poor-house. His conduct in the house was such that no one ever anticipated he contemplated laying violent hands upon himself. On Good Friday evening he took a little walking exercise to improve his health, and an attendant was sent out with him to show him round the neighbouring fields, and in doing so came near the railway just as the down express train came in sight. On hearing the noise of the train he darted from his attendant, and ran upon the line before the engine had come up. His keeper called loudly to him to keep back, but instead of doing so, he went forward to meet the train, and before the momentum of the engine and carriages could be arrested, they came almost at full speed upon the man, who held his head down so low as almost to touch the buffer of the locomotive. He was instantly hurled across the rails, when the wheels of the engine and all the carriages passed over him and killed him on the spot. — Verdict—Temporary insanity.

W—— H——, aged 44 years, committed suicide by throwing himself from a window in the following singular manner, by which he received a hurt on the scalp, and other injuries, which terminated in inflammation of the lungs. It appeared that deceased believed he was entitled to some property which was mortgaged, and he was frequently in a state of great excitement from fancying that he was deprived of its possession. On Saturday night he was in the kitchen of the house where he resided, when he was under the impression that he had seen a ghost the previous day, and asked for a cleaver, with which he said he intended to cut it to pieces should it come. He then observed that the room was on fire, and that he saw the smoke coming through the floor. A female, with whom he lived, endeavoured to persuade him to the contrary, but he became so outrageous that she left him, and remained in the area all night. The next forenoon he suddenly rushed out of the house, crying "murder," and "thieves." He ran up Crown-street, and entered the house No. 2, and passing by a female, the occupier of the shop, ran upstairs, forced open the door of the second floor, into which he went, flinging down a lad in the room. He then went to the window, which was open, and from which he dropped into the street. He was conveyed thence to Charing Cross Hospital, where he died.—Verdict—Temporary insanity.

J—— I—— committed suicide at the Rainbow-hill tunnel, on the Oxford, Worcester, and Wolverhampton Railway. At the inquest the following facts were elicited. The deceased, it appeared, had for some time been in a very desponding state of mind, and on Wednesday went out to be shaved. He was seen in the course of the day by the side of the railway, near the Rainbow-hill tunnel. He was watching the approaching train from Birmingham; and just as it came up, he suddenly laid himself down upon the rails immediately before it, deliberately placing his neck across one of the rails. In another moment, before the driver who saw the act could stop the train, it passed over him, completely severing his head from his body, and carrying it to some distance, while the trunk was terribly lacerated and torn. — Verdict—Temporary insanity.

J—— A—— D—— committed suicide under the following circumstances:—Deceased was 39 years of age, was a civil engineer by profession, and formerly a pupil of Mr. V——. Latterly he became subject to great excitability, and for the benefit of his health had been staying with Mr. R——, of T——. He expressed to this person a great horror of self-destruction, and begged him to take charge of two razors for him. He continued, however, to get worse, and had a strong propensity to throw himself out of window, which he eventually did. —Verdict—Temporary insanity.

W—— H——, 34 years of age, was charged with having stolen a bill, valued at 7*l*. The prisoner, who had a wild, unsettled expression about the eyes, pleaded guilty, and while in gaol he made an attempt to cut his throat with a razor. It had been the practice of allowing the untried prisoners to shave themselves, and while performing this operation the prisoner drew the razor he was using across his throat. A turnkey, who was fortunately standing near him, instantly seized his arm, and prevented him from completing his suicidal intention. He had already been an inmate of a lunatic asylum.

A—— F——, aged 42 years, the wife of a labourer, attempted to commit suicide by cutting her throat. It appeared that for more than twelve months she had suffered from a disordered intellect, and it is supposed than an intimation of being about to be sent to the workhouse preyed upon her mind, and led her, whilst left alone, to attempt self-destruction. She got a razor and inflicted a wound of about five inches in length in her throat; but she did not injure the trachea or any of the larger arteries. The wound being dressed, she was sent to the hospital.

S—— E——, a dissipated-looking man, jumped from off the parapet of Blackfriars bridge, intending to commit suicide, but was saved by a waterman. He was subsequently taken before a magistrate, when his father stated that his son was at times insane, and had been confined in an asylum for six months. His intellects had been impaired by excessive drinking.

J—— H——, 23 years of age, put an end to his existence by swallowing a very large quantity of oxalic acid. It appears the unfortunate man *had been in a very desponding state of mind for some length of time.* He was taken to St. Thomas's Hospital, where the house-surgeon used the stomach-pump and other antidotes, but with no benefit. No cause was assigned for the rash act.

Z—— J—— was charged, at the Guildhall police-court, with breaking a quantity of glass and a portion of the furniture in the house of Mr. E——.

E—— S——, assistant to the prosecutor, who keeps a fancy repository, said he was attending to some ladies, when he heard a violent knocking at the door and ringing of the bell. The housekeeper opened the door, and the prisoner made his way in, and immediately rushed up stairs. Witness obtained the assistance of three constables from the station, but in the meantime the prisoner had locked himself up in the drawing-room on the first floor, and having broken all the glass, he commenced throwing out the furniture from the window, and when the constables arrived he was in the act of throwing himself out. The officer said, that on coming to the assistance of the last witness, he saw the prisoner getting out of the window; he immediately ran upstairs and forced open the door, and was just in time to catch hold of the prisoner by his coat, as he was in the act of jumping from the window into the street. He struggled very violently, but with the aid of the other officers the prisoner was ultimately secured.

Mr. Christie, relieving-officer, said he knew the prisoner well. In October last, he placed him in a lunatic asylum, and in December he was adjudicated as belonging to the parish of S——, and was accordingly removed thither. He was discharged in April last, since which time he had been living with his mother, a very hard-working and industrious woman. The prisoner had previously been confined in the Bethlem Lunatic Asylum, and his father had been similarly placed.

Alderman Salomons ordered him to be taken to the East London Union, under the 16th and 17th Victoria, which provides that wandering lunatics should be taken to the union of the district in which they might be found wandering.

On March 17th, 1853, I—— T——, a boot and shoe maker, residing at No. 4, Princes-place, Clifton, Bristol, destroyed his two children, one aged four and a half and the other six years, and afterwards committed suicide. On the day above named his wife had occasion to go to Bristol, and left her husband at home with her two children. After she had gone, he sent the servant girl out on an errand, and upon her return, finding the door of her room locked in which she had left her master and the two children, she obtained assistance, and upon the door of the room being broken open, it was discovered that the children had been both murdered, and that the father had terminated his own existence. The head of one child was completely severed from the body, and the murder was effected by the means of a shoemaker's knife very much sharpened. T—— had lately been in a very desponding condition, and was heard to express a fear that he would not be able to carry on the business, but he was not in any pecuniary difficulties. At an inquest held on the bodies, the above facts having been proved before the jury, they returned a verdict—That deceased having murdered his two children, committed suicide while labouring under a state of temporary insanity.

J—— M——, a shoemaker, about 57 years of age, murdered his wife, and afterwards committed suicide. The house was tenanted by M——, who with his wife occupied the parlours, letting the remainder out in lodgings to various persons, and one of the upper rooms to his married daughter and her husband. Shortly after eight o'clock on Wednesday morning, Mrs. W——, M——'s daughter, came down stairs, and, not finding either her father or her mother up as usual, knocked several times at the door of the front parlour, occupied as their bed-room, but receiving no answer, she effected an entrance,. and found the place covered with blood, and the lifeless bodies of both her parents lying across each other on the bed, both of them quite dead and cold, and a shoemaker's knife covered with blood was found on the bed, near the right hand of the husband. They were both in their night-dress, and the head of the woman was literally severed from her body. A letter had been written by M——, stating the circumstances which led him to commit the horrible crime. Both himself and his wife were seen and conversed with at twelve on the previous night, and both seemed in their usual health and spirits. An inquest was held on the bodies, when the following was the evidence given on that occasion.

Mary W——, the daughter of the deceased, stated that the deceased was a boot and shoe maker. "I found my father and mother dead on the morning of Wednesday last, in their bed-room, and medical assistance was sent for. I saw my father on Tuesday night, between nine and ten o'clock, and spoke to him, and about eleven I was looking out of the window, and saw my mother coming home, but I did not go down to speak to her. When I saw my father he was in the parlour, and I went down to see how he was, for he had been poorly lately, and complained of pains in his head. He could not sleep well or lie down in his bed for some weeks past; when he complained of his head he felt confused and had strange sensations. His appetite had also failed him lately, but he had not had any advice. He was a very temperate man, and had lately three or four bottles of medicine from Mr. W——, the chemist, in W—— street. I have noticed my father apparently bewildered lately. I never heard him threaten to destroy himself or my mother: quite the contrary." A letter, identified by the daughter as the handwriting of her father, which was found upon the mantelshelf, and addressed to Mr. ——, the landlord, was read, of which the following is a copy:—

"March 28, 1854—9 o'clock, p.m.

"Mr. C——:

"Sir,—I am extremely sorry that the settling of my rent to-morrow will be in a way you little think of, which must be by your taking my goods, and I hope you will find sufficient to pay you. I find it impossible for me any longer to work so as to be able to keep myself, and I have no wish to live on the labour of others. The asthma which I am troubled with precludes me from working to do any good, more than the few warm months of summer. My eyes are likewise failing very fast, so that I have made up my mind to leave this world. And as you know the dreadful state of mind my wife is sure to be in if I leave her, I have made up my mind that she shall go with me.

"To my children.

"I dare say you will censure me for the rash act which I am about to commit; but if you think it over seriously, you must know that your mother will be better off a great deal than being left behind me. Hoping you will get over the shock with as much firmness as you can, I remain, dear children, your affectionate father,

"J—— M——.

"To Mr. Carter—I remain, sir, yours most respectfully, and sincerely thank you for all favours.—J. M.

"To my very particular friend Mr. W——, of W—— street—Good bye.—J.M."

Mary W——, the witness, then continued, who further stated: I have often seen him resting his head upon his hands, and heard him complain of his head. He was on the most friendly terms with his landlord, Mr. C——. He was not in want of work. My father and mother were living upon the most happy terms. He never used her ill in any way. She was a very sober person, and as kind to him as he was to her. I don't know that he was in difficult circumstances. He was two quarters behind in his rent. He has held the house nineteen years. He was not a proud man, but quite the reverse.

Mr. I—— N—— J——, surgeon, stated that he was called to see the deceased persons about half-past eight on the Wednesday morning, and found them in bed with their throats cut, and the smaller knife grasped in M——'s right hand, and the larger knife lying near the bottom part of the bed, on the wife's side of the bed near her feet. There was no blood flowing; the bodies were stiffened, but not quite cold; the bed-clothes were not in the slightest degree disturbed, indicating not the least struggle; the only unnatural position was the elbow of Mr. M——, which was placed over the throat of his wife. I did not notice that the upper part of the wife's bed-gown had been cut; the wounds were very large, and the principal vessels of the neck, in both cases, divided. The woman's throat was cut down to the bone—to the vertebræ—those wounds were the cause of death. I have no doubt that the man made both the wounds; it is impossible from the character of the wound in the wife's throat that she could have inflicted such a wound herself. I believe the wounds were inflicted in the night.

Emma S——, a lodger in the house, stated that she thought M—— lately to have been more dull than usual. He was very dull on Tuesday night, and said but very little; had heard him say he could not sleep or rest on his bed. Between ten and eleven o'clock on Tuesday night, he fancied he heard a knock at the door twice, and that it was Mrs. M——, and he went to the door, but there was no one there. When Mrs. M—— came home, she was very full of spirits, and she came and spoke to me in my room, which was next to that where the bodies were found. I saw Mr. M—— write a letter like that produced, and he held it up to me, and said, "There, do you think that will do?" I said, "I don't know, is it going to a young woman?" and he replied, "Oh no, nothing of that kind."

George M——, son of the deceased, stated that he worked with his father in the same room, for company's sake. I worked there on Tuesday morning, and had remarked a great change in him lately. He was an industrious man generally. He told me he was prepared for his rent. I am not aware of any circumstances lately to disturb his mind, but I have heard him say he should be sorry to be a burden to any one. I never knew him the worse for drink, or to have any words with my mother. He was a man very fond of reading, but his sight has been getting bad for the last three or four years.

The coroner, Mr. Wakley, expressed a wish to have the deceased man's head opened, as he thought that sufficient disease would be exhibited to show that when he committed the dreadful act he was in such a state of mind as not to be responsible for his own actions. A most important lesson, it appeared to him, would be taught the public by this inquiry. Here was this poor man for months with a pain in his head, showing that his brain was affected, and he could get no sleep, and yet he ceuld get no medical treatment, except what he obtained from a chemist and druggist, who probably knew nothing at all about what was the matter with him. It was a remarkable thing that so little attention was paid to the head, which was the principal organ of our system; persons would attend to an arm or a toe if it was diseased, but not to the head; that disease was allowed to creep on in the head until it rendered the state of a man's mind such as to lead to deplorable acts like that they had met to investigate that day. He felt firmly convinced that had this poor man had proper medical treatment six weeks or two months ago, he and his wife might be alive at this time.

It was decided that the head of this unfortunate man should be examined, to ascertain, if possible, by such examination, what was the probable state of his mind.

*Mr. Wakley added that he had no hesitation in saying that six out of every seven cases of suicide might be prevented if, when a person loses his sleep and becomes first attacked with unusual pains in the head, he immediately sought medical advice.*

The head having been opened by Mr. J——, the surgeon, he stated : I found the dura mater very much thickened and inflamed, and the pia mater and arachnoid also inflamed—the brain itself was very healthy, but injected. I should say that the brain had become injected within the last three months. If the man had sought medical advice, the treatment would have been either blister or bleeding about the head, and medicine of an aperient character. The ventricles were devoid of fluid. There was quite enough disease to account for pain in the head. There was no disorganization of the brain.

It appeared, from the statement of one of the jurors, that the wife had been insane, and in a lunatic asylum, and he thought she was as likely to cut her husband's throat as he was her throat ; but Mr. J—— said that, from the position of the bodies, that was impossible.

*The coroner, in summing up to the jury, repeated his conviction as to the necessity of immediate medical advice being resorted to in all cases of pains in the head or disturbance of sleep.* The law held homicide to be murder ; and in this case, if the jury believed the deceased to have been in a sound state of mind when he committed the act, they had no alternative but to return a verdict of wilful murder, and felo de se ; but he thought, after the evidence showing there was active and extensive disease of three of the membranes of the head and brain, there was no doubt that when the deceased man committed this act he was not a responsible being.

The jury returned a verdict—That the deceased, J—— M——, deprived his wife of her life by cutting her throat at a time when he was labouring under an unsound state of mind, and also deprived himself of life while in the same state of mind by similar means.

It subsequently transpired that the wife had been afflicted with insanity, and twice been an inmate of the lunatic asylum, but had latterly become perfectly restored, and was an exceedingly industrious and well-behaved woman, aiding her husband by going out charing in the neighbourhood. Doubts were entertained whether she was not herself a party to the horrible crime her husband committed, and a very remarkable fact was also stated by Mr. C——, the landlord of the house, that M—— himself, about twelve months since, in a conversation with him, informed him that his wife had made a proposal to him to murder her, and then destroy himself, so that having lived so long together, they might die together; adding that she could not live in the world without him. This conversation is taken to account for the remark made in the letter written by the unfortunate man the evening before he committed the crime, addressed to Mr. C—— : "and as you know, sir, the dreadful state of mind my wife is sure to be in if I leave her, I have made up my mind that she shall go with me."

G—— W——, after attempting to murder his wife, committed suicide by cutting his own throat from ear to ear. He had evinced a slight aberration of mind for some days past, but retired to rest with his wife on the Sunday evening, apparently much better than he had been. Nothing particular occurred until after five o'clock on the Monday morning, when his wife was awoke by his attempting to cut her throat with a razor. She rushed from the room to procure assistance, and upon her return her husband was found a corpse, having nearly severed his head from his body.—Verdict—Temporary insanity.

B——T——H——, a tin-plate worker, residing at 15, K—— street, S——, 34 years of age, committed suicide by cutting his throat with a knife, after a desperate attempt to murder his wife. For some time past he had treated his wife unkindly, and it is supposed that latterly he had entertained suspicions, which continually haunted his mind, that his wife was not faithful to him. On Wednesday, December 21st, 1853, before the attempted murder was committed, his wife had been confined, and ever since the birth of the child it had been the subject of repeated taunts from her husband. When she was confined, he came and looked closely at the infant, shook his head, and said, "Enough, enough." His wife's mother had come up from Lancashire to nurse her daughter in her confinement, and her presence operated as a considerable restraint upon the ill-treatment of H——. On the Saturday evening, the 24th, he taunted his wife about the child, but afterwards became more calm, and took the mother-in-law out, with a view, as he said, of purchasing some necessaries for the infant. After having been out some time together, he escaped from the mother-in-law, and returned home in a frantic state, dashed into his apartment, and with a sharp-pointed Italian spring knife open in his hand, he furiously stabbed his wife as she was lying on the bed with her infant, without making any observations, inflicting several severe wounds upon the fleshy part of the right arm and side. When found by the police, H—— lay partly on the bed, with his head nearly severed from his body. The carotids on both sides were literally cut through, and so were all the structures of the neck down to the cervical vertebræ. It was an unusually extensive wound for a self-inflicted one. He was quite dead. The wounds on the wife were no doubt inflicted with the knife, while his own throat had been cut with a razor, which was found in the bed-room smeared with blood. He had purchased the razor last summer, and in a quarrel with his wife stated that they should both go off together in one bloody bed. His wife was 27 years of age, and had been married only eleven months.—Verdict—Temporary insanity.

W—— T—— made a most determined but ineffectual attempt to murder, followed immediately afterwards by the suicide of the young man himself.

It appeared from the evidence at the inquest, that Miss W——, a young woman, proceeded to S—— street on the Monday morning to collect the rents of houses belonging to her father, and in doing so she entered the dwelling of T——'s mother, a widow and monthly nurse. Mrs. T—— paid the rent, and Miss W——, after entering it as usual in her book, addressed the deceased, who was moodily pacing the room, inquiring after his health, which had been declining. He replied he was not much better, and then suddenly advanced to where she sat behind the door, and raised his hand as if to strike her. Miss W—— screamed, and T——, with a razor he had hitherto concealed, instantly inflicted two wounds on her neck, and a third on her shoulder, besides cutting her dress in several places. His mother now sprang forward and seized his arms from behind, but the unhappy man stooped his head, and his throat coming in contact with the razor, he inflicted a deep wound which severed the principal veins and arteries, and resulted in death half an hour afterwards. Two neighbours, attracted by Mrs. T——'s cries, entered the house, and having removed Miss W——, took the razor from the hand of T——. Upon the arrival of the surgeon, Miss W——'s wounds were sewn up, and prompt assistance rendered to the deceased, but he expired, declaring himself poisoned. It appeared that for some weeks past T—— had been unemployed, and during that time he had been subject to monomaniacal belief that his food was poisoned, refusing to touch it until his mother or sister had previously partaken of it in his presence. In consequence of these delusions, he was examined by Mr. Freer, surgeon, who recommended his removal to a lunatic asylum ; but his mother, believing his insanity to be merely temporary, neglected to follow the advice.

After a lengthened inquiry, the jury returned a verdict of insanity. His mind was said to have been impaired some time past.

N—— S—— was a basket-maker, residing at B——. He had three children, two boys and a little girl, all by a former marriage. The boys were aged, one eleven, and the other was in his seventh year. On Wednesday morning he went out and took the two boys with him, but did not assign any cause for doing so ; he also wanted to take the girl, but his wife sent word to the governess where she was at school not to let her out. The youngest boy, who was also at school, he called for himself. The wife begged of him and entreated him not to take them away ; but pushing her away from him, he told her she should not see him again, but would hear from him in a few days. On the following morning the widow received a letter from her husband, of which the following are the contents :—

"By the time you receive this, me and my boys will be locked in the arms of death, and I am very unhappy that the girl is not with us. You have to thank your own temper towards me, and I made up my mind on my pillow this morning what I should do before I started ; but I have little comments to make, but your temper has been that to me, that it has preyed on my mind for some time ; but it is finished before this time, and I hope that my girl will grow and be a good girl, and I should have been happy to have had her with us, and I hope that you will govern your temper for the future. You have none to thank but yourself for this, and I hope that you will do well. God bless you both for ever. Amen. M. S."

The following day the bodies of the man and the two boys were all found drowned in the river Thames, between P—— and W——. The body of the man was floating by itself, and the bodies of the two boys were fastened together by some stout twine, formed into loops round the waist, and a longer piece encircled them both together. The boys were tied about a foot apart, there were no marks of violence about them, nor any handkerchief nor anything tied about their eyes. The man was tied round the arms by a cord, but not so close as the boys; his legs were fastened by a stout willow, which was again crossed by another, as well as one twisted very tightly round his throat, nor had any of them either a cap or a hat on. When the bodies were searched, there was nothing found that could show the cause of death. The boys were tied face to face by four lines of twine, which were knotted quite tight, and the withes round the man's throat and ankles were fastened very tight indeed. They were all met on the previous evening about half-past seven o'clock, on the road from P—— towards H——, when they all appeared very cheerful, and the two boys were playing along the road with sticks and the bung of a barrel.

The widow of the deceased stated that they had been married eighteen months, and that about two months after that event the husband tried to cut her throat, and she was obliged to put both razors and knives away. He was addicted to drink, and during such times he was nearly mad. I am certain he was in a deranged state of mind. He treated me very cruelly, and often beat me. On one occasion he took the children out of their beds at three o'clock in the morning to destroy them, but I prevented him.

It also appeared, from the evidence of the apprentice, that when sober, deceased was fond of his children and spoke very highly of his wife, but when intoxicated he was very bitter against her. Business had much fallen off lately, and he was much beside himself. He was much embarrassed, and had no capital to execute an order which he had received, which preyed upon his mind, and he told the apprentice he should not mind if his time was come to walk the plank and die. His wife had always behaved with propriety, but he often raised a quarrel with her for no cause.

Verdict—That the two boys, N—— J—— S——, and W—— S——, were wilfully murdered by their father, who afterwards committed suicide by drowning, while in a fit of temporary insanity.

J—— D——, gardener, murdered his wife and child, seven years old, and committed suicide by drowning. About a hundred yards above the spot where the bodies were found a large clasp knife such as is used by gardeners was discovered. The wife and child were found in the cottage of deceased lying on the floor with their throats cut, so that their heads were nearly severed from their bodies, the furniture of the room much disordered, and from the marks of blood on the several doors and the wounds inflicted on the woman, it was evident that a dreadful struggle had taken place between her and her murderer. The woman and child were both in their night dresses, from which it was evident that the attack upon them had been made after they had retired to rest, and by marks of blood being found upon the bed of the latter. The body of the woman presented an awful sight, having a deep incision in the neck, extending obliquely from the right side below the throat to the back of the neck on the left side, dividing the muscles, vessels, windpipe, and gullet, notching the front of the spine, and completely severing the lateral bony process of the spine. It was a double cut, one superior and superficial—the second, upwards of six inches in length, down to the muscles extending from the right shoulder, and terminating on the collar bone. There were other wounds on the neck and other parts of the body, with a large bruise and an infusion of blood on the left temple. The lad had received a deep gash, confined principally to the right side of the neck, dividing muscles and vessels, and penetrating between five and six inches to the spine, and partly severing the head from the body; the cut commenced at the back of the neck and extended obliquely downward to the top of the breast-bone.

It appears that he had for the last few days been labouring under great depression of spirits, to which he had been previously subject, and that after committing the crime he attempted to hang himself in the green-house.

D—— had for a number of years, at different times, suffered from mental debility, so much so, as at times to appear like a madman. He had left several situations in consequence of the diseased state of his mind, but all was kept secret from his employer, to whom he had for three years been a faithful servant, and so much so, that he had consulted his medical man, Mr. Cotton, on the previous Monday, to attend him, which he did, but did not consider him in a bad state, although he had afterwards recommended that he should have a change of scene and air, in the hope that it would be the means of recovering him from his hypochondriacal state of mind, which would soon degenerate into insanity, so as to cause him to commit some dreadful crime.—Verdict—Insanity.

Mrs. G—— murdered her child, and subsequently destroyed her own life by drowning. The infant was only four months old, and the bodies of both the mother and child were found drowned in the water-butt. Dr. Marsh promptly attended, and used every means to restore animation, but without success. The mother was represented to have been a well-conducted, sober, and industrious woman, but having been in ill health *ever since the birth of the child, that circumstance is supposed to have preyed on her mind, and produced despondency, which had prompted her to the perpetration of the murder of the child, and the subsequent destruction of her own life.*

The jury returned a verdict—That the deceased infant was wilfully murdered by her mother, being at the time in a state of temporary insanity.

An inquest was held on the bodies of Mr. A—— N—— and Mrs. H—— N——, at the residence of the deceased. Mr. N—— was an agent, well known as one of the largest shipping merchants. Mrs. H—— N—— was his sister-in-law, the wife of his late brother, L—— N——. Mr. A—— N—— was a widower.

It appeared from the evidence, that both Mr. and Mrs. N—— attended church on the Sunday morning, and again in the afternoon of the same day. That they afterwards accompanied a Mr. C——, one of the executors of the late Mr. L—— N——, and husband to the deceased lady, to his house to tea, and returned home early in the evening. The servant, at the request of Mrs. N——, brought two tumbler glasses and a jug of hot water before retiring to bed. The next morning the boy went into the dining-room to open the shutters, when he found Mrs. N—— lying dead on the floor. The servant-man instantly proceeded to Mr. N——'s bed-room, when he found him suspended, by the cord of his dressing-gown, to the mahogany bar of his bedstead, with his knees touching the floor. The bed was quite warm, as was also the body.

Mr. H——, a surgeon, stated that he had made a post-mortem examination of the bodies, and that upon the head and windpipe of the deceased lady there were marks such as might have been made by finger-nails, and that those marks corresponded to ecchymosis. Immediately underneath, the great vessels were gorged with blood, and on opening the chest he found the lungs gorged with venous blood, and the right side of the heart also. There was nothing found in the stomach; it was in a perfect state of health, and free from any appearance of poison. The head and membranes of the brain were highly congested. The condition of the head, lungs, and heart was quite sufficient to account for death, which arose from strangulation; there was nothing else to account for the death. On the nose of Mr. N—— there was a bruise, such as might be caused by a scratch, which it was supposed he might have got in an encounter with the deceased lady. Mr. N—— was about 28 years of age, and Mrs. N—— about 30, and had had two children, and she had resided with Mr. A—— N—— since her husband's decease.

Mr. J. H. H——, jun., who acted as solicitor to the executor of the late Mr. L—— N——, deposed that he had been in the habit of seeing Mr. A—— N—— on business matters; that he had one or two interviews with him on the 15th, and again on the 26th of December, and several times afterwards. That Mr. N—— had said upon one occasion, when going through a number of accounts, "*I must give up this work; it makes my head feel bad, it makes me ill, and confuses me;*" and

*on two or three occasions after the 26th of December last, he said, "Well, this business upsets me; I hardly know what I am doing, my head is so bad." He also on one occasion said that the medical man had told him that he must not exert himself too much, that it would make him ill. On another occasion, having some conversation as to the transaction with the business at the Stamp Office, he became cross and irritable, more so than witness had ever seen him before,* and that while witness was adding some accounts, he made a remark which he could not catch; and upon asking him what it was he said, he made no reply, but continued staring at the fire, and did not look up. Without speaking a word, he jumped up from the chair, slipped on his coat in a minute, and bounced out of the room without saying a word. Witness further stated, "I remember him at school, some nineteen or twenty years ago, and he was always looked upon as a weakly, soft-headed boy, compared with his brothers. He was decidedly passionate and impulsive, and did not control himself."

Mr. C—— H—— B—— confirmed the evidence given by Mr. H——, that he was extremely irritable, and excited by the least thing. He was a temperate man. About seven or eight years since he had been in business in L—— on his own account, in partnership with one or two other persons, since which time he had not been in any business.

Mrs. N——, his mother, was insane, and was confined for some years, and two brothers also died by their own hands. A sister of his at the present time is not in a sound state of mind.

Mr. Harrison, the surgeon, said that not only would it be possible, but extremely probable, that such a person as Mr. N—— had been represented would, under extreme excitement, be betrayed into maniacal impulses. There are many cases of this nature. People who are capable of pursuing the usual transactions of life without showing any external appearance of insanity, may, from a sudden impulse, commit either great crimes or some act that would fall under the common denomination of insanity.

Verdict—That Mr. N—— destroyed the lives, first of Mrs. N——, and afterwards of himself, being at the time of unsound mind.

A Mrs. S——, with her son, S—— S——, a young man little more than 20 years of age, an engraver, who is stated to be somewhat weak in his intellect, resided with her brothers, Messrs. J—— and T—— D——. The youth, it appears, had lately been depressed in spirits, in consequence of a slackness of work, and on Saturday morning, the 18th of March, he went to the house of his cousin, J—— P——, a pistol finisher, and asked leave to cast some bullets to fit a small pocket pistol he had borrowed from a companion with whom he had been out shooting. He found, however, that the mould would not fit the pistol, and having cut up some lead into slugs walked away, remarking that he would go and have a good shot, as there was some waste ground near his dwelling. Shortly afterwards he returned home and breakfasted with his mother, one of his uncles, his niece, and his grandmother. After breakfast he was left alone with his mother, and directly afterwards, hearing the report of a pistol, M—— D——, the niece of Mrs. S——, rushed into the room, and found her aunt falling from the chair covered with blood, her son, S—— S——, standing beside her with a pistol in his hand. The girl exclaimed, "Oh, S——, what are you doing?" he made no reply, but turned and looked at her for an instant, and then ran upstairs. Mrs. S—— lay upon the floor, with the blood flowing from a dreadful wound in her head, and the cries of the terror-stricken niece on beholding a sight so appalling brought several persons to the spot. Shortly after, another report of fire-arms was heard upstairs, and the youth who had thus murdered his mother was found dead by his bedside, shot through the head by his own hand. Death, in both instances, must have been instantaneous. It is supposed that the fear of seeing his mother reduced to want had preyed upon his mind, and had prompted him to the commission of the murder. The mother was 50 years of age, and respectably connected.

C—— B—— was tried at the Huntingdon assizes, March, 1849, before the Lord Chief Baron, for the murder of his daughter, a child two years of age, by cutting her throat with a razor.

At the P—— summer assizes, in the previous year, the prisoner was tried before Mr. Baron Parke for the murder of his wife. On that occasion, as now—both the lives having been taken away at the same moment almost—the defence relied upon was insanity; but the jury finding the prisoner guilty on the former occasion, he was left for execution. Further inquiry, however, having been made into the state of his mind, he was reprieved by the Secretary of State, in order that he might be put on his trial for the murder of his daughter, and an opportunity thus afforded for the production of an additional evidence which might be obtained in the meantime.

The evidence now adduced for the prosecution was to the effect that the prisoner, on the day before the death of his wife and child, took the latter with him to a neighbouring barber, when she sat on his knee while he was being shaved. That day he directed a razor to be ground for a lodger. At four o'clock in the morning, a noise was heard in the prisoner's house, and when his next-door neighbour got in he found the prisoner standing in his shirt, bleeding at the throat, his wife and child lying dead at his feet, with their throats cut nearly in two. The prisoner was asked by his neighbours as to the death of his wife and child, of whom he was particularly fond, but he said nothing until he saw Mr. N——, a surgeon, who, on arriving, said to him, "Good God! B——, what have you done?—had you and your wife quarrelled?" The prisoner only shook his head, his wound preventing him making any articulate reply. When asked if he had been in any trouble, he nodded. In a little time he got better, but remained moody and depressed, till at length, in answer to the question, "Why did you do it?" he said, "Trouble made me do it." He was then asked when he first thought of it, to which he replied, "only at the moment when I got up to destroy myself; I had the razor sharpened to destroy myself, and had contemplated doing so for a week, during which I had pains in my head. I did not sleep that night, and got out of bed to destroy myself, when a thought came across me that if I did, my wife and child would come to want when I was gone. I then instantly attempted to cut my wife's throat; she was partly asleep; when I attempted to do so, she jumped out of bed and rushed to the window, shrieking 'murder.' I then cut the child's throat, and went to the window, where I took my wife in my arms and threw her on the floor backwards; I then pulled back her head and cut her throat a second time, which destroyed her, after which I attempted to cut my own throat, but could not carry it out."

When cross-examined, Mr. N—— stated that he did not think the prisoner was in a sound state of mind at the time he did these acts, and there was nothing in his subsequent communications with the prisoner which would lead him to alter the opinion that he was insane, though he observed no delusion.

In order to prove the prisoner's insanity, Ann Jordan, a daughter of the prisoner's grandfather by a second wife, stated that one of her (witness's) sisters was now insane, and another had cut her throat.

Matilda Gorham stated she had known the prisoner all her life—he was a cheerful man, but the week before the murder she observed a change in him.

F—— B——, the brother of the prisoner, stated that, as he had heard, his uncle John, who is dead, was insane.

John Wise stated that he had seen the prisoner soon before the murder, and thought him strange, inconsistent, and altered from what he had been; he thought him in an unsound state of mind then.

Mr. Z——, a surgeon, stated that he had seen the prisoner before the murder, and observed a gloominess and silence in him. He saw the prisoner on the Monday, when he was suffering from mental depression, he then thought him of unsound mind, and thought him so still. He said, "I was not surprised at the murders, for I fully expected he would destroy himself. The countenance will betoken the state of the mind." Upon his cross-examination, he added, "I believed he was a lunatic when I saw him. He was dealing in bones and rags to the last, and neglected to bring me a grate from P—— for some weeks."

George Smith, the governor of the gaol at H——, stated that the prisoner had been in his charge since the last assizes. "He has shown symptoms of insanity, and I treated him as an insane man. He once told me that a cat had been thrown on him to tear him to pieces, and that he had been put under a cart to be killed.

He received the announcement of the day fixed for his execution, after the last trial, with unconcern, and has always told me he has committed no offence, and has treated the deaths of his wife and child without any concern. I have always kept two men in the room with him, and think he was, and is, of unsound mind. I am sure that his feelings expressed to me were real, and that he practised no dissimulation. I was aware that he had attempted suicide before he came to the gaol.

Evidence was then given to show that the prisoner was not insane, and it was stated by a Mr. G—— W——, a surgeon, that he saw the prisoner on the day before the death of his wife. I observed no change in him. I spoke to him, and have been in the habit of seeing him two or three times a day. I do not think, as far as my judgment goes, that the existence of madness is to be inferred from the facts proved, but they are not inconsistent with mental disease.

John Cobley, a cousin of the prisoner's, who had known him thirty years, stated that he went with him to P—— on the 27th of May. On our way home he called at Mr. M——'s, and got a razor. I stayed with him till half-past seven o'clock, and saw nothing peculiar in his manner throughout the day. I decline to say whether I have myself contemplated suicide.

Mr. N——, the surgeon, positively swore that the last witness had spoken to him in the manner and terms denied by him.

The Chief Baron, in his summing up, said the question for the jury was, whether they thought the act of cutting his child's throat, under the circumstances proved to them, was the act of a desperate and wicked man, conscious of what he did, and regardless of the lives of others and his own, or whether it was the act of a man oppressed by disease which affected his mind to such an extent that, while it left him some rays of reason, rendered him a person of unsound mind. The plea of insanity could only be supported by proof of positive disease. The act itself ought not to be taken as a proof of insanity, and it was a matter of surprise that no medical man had been called who had observed the prisoner's conduct while he was in gaol; such testimony would have been more satisfactory than that of the gaoler, and the jury must form the best conclusion they could with that evidence which was before them on this case, the result of which would deeply affect the public. Atrocity was not to be taken for insanity, but at the same time there could not be a more dreadful spectacle than the execution of a man who was guiltless of crime before God.—Verdict—Not guilty, on the ground of insanity.

M—— J——, aged 23, was indicted for the wilful murder of her own child, E—— J——, and her niece, M—— S——, at the town of W——. This double murder was committed under the following circumstances:—

The prisoner was the wife of one E—— J——, and had been married about two years, and had the child named in the indictment, who was eighteen months old in August last. There was also living with her husband and herself the little girl, M—— S——, about 12 years old, the daughter of one of her sisters.

Between the prisoner and her husband there had always existed the best feeling, and towards her own child she had never, before the murder in question, been observed to act otherwise than with affection, nor was there anything to show the slightest ill-feeling against the little girl. It appeared, however, that for about eight months before the 21st of August last (the latter end of 1847), the manner of the prisoner became strangely altered, and she had threatened to burn her child. She had been brought up a Protestant, but just before the change in her manner was observed, she had been in the habit of reading Roman Catholic books, and used to attend Roman Catholic worship. She had had her child also baptized by a Roman Catholic woman after it had been baptized by a Protestant clergyman. Subsequently, however, she used some expression of regret about the child's baptism, and she thought it would go to hell, and desired that the incumbent of the parish might be asked to baptize it again.

It was proved, further, that she laboured at times under the delusion that her mother (who had been dead eight or nine years) was haunting her, sometimes sitting beside her, and at other times following from behind. She was subject, too, at intervals to severe paroxysms of excitement, during which her violence was so great as to render it necessary to hold her by force. Her friends obtained from a surgeon a certificate of her being a lunatic, and she was admitted into the W—— Union workhouse, preparatory to her being sent to the county lunatic asylum. In the workhouse she was put in the insane ward, and exhibited the same illusion and the same violent paroxysms as she had before shown; and the surgeon of the union, upon his examination, stated that he also considered her of insane mind, subject to lucid intervals. By the 13th of September, as appeared by the same surgeon's evidence, she had become better, but, in his opinion, not in a condition to be left alone; and having no power to detain her at the workhouse, it not being a licensed asylum, he allowed her to leave the workhouse, and return home with her friends, who came for and who took her to her own house, and most imprudently left her with the two deceased children, her husband at the time being from home.

About eleven o'clock the same night some of her neighbours were alarmed by shrieks proceeding from her house, and upon going thither discovered that the shrieks proceeded from her. She was discovered standing in her bed-room window in her night-dress brandishing a knife, and she was heard to call out several times, "I have cut their throats!—I have cut their throats!" Several people then entered the house, the unfortunate woman's brother being one of the number, and the first to enter. On seeing her brother she called him by his name, and said, "I have cut their throats—come and cut mine;" at the same time drawing the knife which she had in her hand across her own throat. Her brother immediately rushed upon her and secured her hands, but not before she had succeeded in inflicting two severe wounds on her throat. He then gave her into the keeping of other persons present, and upon going to the bed in the room, the most horrible sight presented itself: the head of the elder child was hanging from the side of the bed, and attached to the body by a mere integument. He then turned down the bed-clothes, and there saw the younger child's body and head, the latter completely severed from the body, and lying beside it. Under the bed and about the room, as the witness expressed it, there was "a lake of blood." The unfortunate woman was then taken off in custody, and to the constable she minutely described how the dreadful deed had been accomplished with the knife she held in her hand. She said that having failed to find her husband's razor, she took a common knife, sharpened it, and cut the "poor things'" heads off—the youngest first; and then added, "I am all right; take me to S——. She was seen soon after by one of the surgeons before spoken of, and he and all the witnesses clearly proved that then, as well as for a period of eight or nine months before the dreadful occurrence, she had been of insane mind, subject to lucid intervals. It appeared also that while in the workhouse she had suffered from a severe attack of smallpox, the effects of which were such as to affect the brain.—Verdict—Not guilty, on the ground of insanity.

T—— W——, aged 45, was indicted for the wilful murder of his wife, E—— W——, at B——. The following are the facts of this case:—

Mary Price, an elderly woman, a cousin of the deceased, went to the cottage of the prisoner, as she was frequently in the habit of doing, and found the prisoner and his wife at tea, and, at the invitation of the prisoner, she joined them, and he placed a cup and saucer for her. About seven o'clock she left, promising to call the following morning to send some things to the Haymarket by the deceased, who expressed her intention to go there. On the next morning, about ten, she again went to the prisoner's cottage, but found the door shut; she tried the latch, but could not open the door; she called out, "Betty! Betty!" and then she heard the prisoner's voice inside saying, "Betty is dead in bed; she cannot come to you." The woman then desired the prisoner to open the door; he replied, "I cannot do so—I cannot find the key." She then went away to the nearest neighbour's, and after an absence of two hours, returned with two men, named Powell and Clerk, to the prisoner's house. The door was still closed and fastened inside. Powell called out to the prisoner to open it; he said, "I cannot find the key." Powell told him he must force the door open, and soon after the prisoner opened it, apparently forcing the lock to do so. They then went in and found the deceased lying on the floor nearly naked, quite dead, her head and skull being beaten to pieces, and the blood and brains scattered about the floor. Pieces of a broken ladder lying near the body bore the marks of having been the instrument of death. When these persons went into the house, the prisoner went out and walked about in front of it.

The woman, Mary Price, stated that on the previous evening the prisoner exhibited a strangeness of manner—that he would not eat at tea, and that the deceased restrained him from drinking his tea hot. The deceased had complained to witness that her husband was in an odd state of mind, the same as he had been fifteen years before; that they, prisoner and his wife, always lived on friendly terms; that he was a good husband, and she a dutiful wife.

Two other witnesses corroborated the evidence of Mary Price, and stated further that the prisoner had said, "I would not have killed my poor wife, but I thought I was killing the great goddess Diana;" and that when he came out of the house after the murder, he read aloud a part of a chapter in the Bible and a psalm. The prisoner used to be employed as a drover, and had been absent from home a month a short time before the murder, having only returned a day or two before. It was also further proved by the policeman who took the prisoner into custody that he said he "thought it was the goddess Diana in the bottomless pit he was killing."

Mr. W——, surgeon, spoke to the examination of the deceased, and to the probability of the death having been occasioned by the blows inflicted with the broken ladder, and stated that he had spoken to the prisoner at the coroner's inquest; that he talked incoherently, but he could not express any opinion as to his sanity otherwise than at that time.

The daughter of the witness Mary Price deposed to conversations, about the 25th October, with the deceased as to the state of the prisoner's mind, in his presence, and also to the odd manner exhibited by the prisoner at the same time; and that the deceased said her husband "had a spell, and she must get it removed, cost what it might."—Verdict—Not guilty, on the ground of insanity.

A woman named B—— was charged with the murder of her two children by throwing them into the canal. She had been committed to prison, and was brought to the court two years since, but was at that time too insane to plead. At the summer assizes she was again put to the bar, it being then supposed that she was so much better, and therefore able to plead and be discharged. Seeing so many people in court, and the naming the offence when she was arraigned, had such an effect upon her, that it became necessary to remove her instantly from the court. At the assizes, she was again brought up, and appeared quite calm and collected; and when she was called upon to plead, she said she was "not guilty." No evidence being offered against her, she was given up to her husband.

M—— P——, a married woman, 42 years of age, an inoffensive, motherly looking woman, attired in deep mourning, was indicted for the wilful murder of S—— S—— P——, her daughter, by cutting her throat. The prisoner had been previously arraigned at the Lent Assizes, and was then found insane and unfit to plead, and since that period she had been confined in gaol, but having now recovered, was placed on her trial. The question for the jury to decide was, as to the state of mind of the prisoner at the time she committed the act imputed to her. The prisoner was the wife of a labouring man near C——, and had always borne the character of an affectionate wife and mother. In 1849 she was delivered of the child in question, and it appeared that shortly after her confinement she was observed to be in a very low and desponding state, and was frequently heard to exclaim she did not know how she should be able to live, as her husband's wages had been reduced. Soon after this her husband was discharged from his employment, and this preyed heavily on her mind. On the morning of the day named in the indictment she went in her night-clothes to the house of a neighbour, named Cook, a shoemaker, in a wild and excited state, and exclaimed to him,—"I have killed my poor dear babe, and have tried to kill myself, but I can't." Upon Cooke saying he hoped it was not true, she told him it was, and put her hand up to her throat, when he observed blood oozing out between her fingers. Upon going to the house the child was found quite dead, covered with the bed-clothes, and the razor with which the act had been committed lying by its side.—Verdict—Not guilty, on the ground of insanity.

H—— R—— was indicted for having at A——, in the county of C——, wilfully murdered her new-born female child. It appeared that the prisoner had lived in the service of Mr. H—— P——, a farmer at Sidehouse, A——. On being charged by her master with being in the family-way, she denied it, but it afterwards transpired that she had been delivered of a child. She was said to have been a good, kind-hearted girl whilst in his service, but was subject to great depression of spirits, and often in deep studies. The child was suddenly taken ill, and died the same evening. Five witnesses were called to speak to the prisoner's character and habits, all of whom said that she was frequently in a low state of mind; and Mr. W—— M——, a surgeon, who had attended her four or five years after a previous confinement, stated that she had suffered from puerperal insanity, which lasted for a fortnight, a disease to which women are sometimes subject, caused by a suppression of milk. Having suffered from it in her first confinement, she would be more disposed to another attack at a second confinement, and being left alone, or being in a desponding state, would dispose to it. She had poisoned the child by giving it laudanum.—Verdict—Not guilty, on the ground of insanity.

J—— I——, the prisoner, charged with feloniously cutting and wounding his mother, with intent to murder her, was 35 years of age, and the only question for the jury to decide was, whether at the time the act was committed the prisoner was in such a state of mind as rendered him legally responsible for his actions.

It appeared from the evidence that the prisoner lived with his mother at N——, and they were on the most affectionate terms. On the Sunday, the day on which the prisoner committed the offence, he, without the least provocation, seized a razor, and attacked his mother with it, and inflicted a most severe injury upon her throat. He then rushed into the street, exclaiming, "I have done it—I have murdered my mother." The prisoner was taken to Horsemonger-lane Gaol, where he was in a raving state of madness, and although he somewhat recovered afterwards, he relapsed upon being removed to Newgate, and Dr. McMurdo, the surgeon of that prison, stated that he was decidedly insane, and incapable of distinguishing right from wrong. It was also proved that it had been necessary to restrain him some years ago, and his mother had been recommended to place him in an asylum. —Verdict—Not guilty, on the ground of insanity.

T—— C—— W——, tried for the murder of his mother. From the evidence of Mr. J—— C——, the uncle of the prisoner, and proprietor of a public-house at D——, in the county of M——, taken before the magistrate at L—— police-court, it appeared that the mother of the prisoner resided at No. 1, Durham-place.

He stated that he was perfectly aware of the prisoner's insanity, and had taken him himself to the Bethlem Hospital; but nine months afterwards he was discharged from there, though in an uncured state, at the earnest entreaties of his mother. After three or four months, however, he was obliged to be sent to the W—— Lunatic Asylum, and there he remained some time, when, at the urgent entreaties and solicitations of his mother, he was discharged. Before his discharge, the committee of the institution remonstrated with his mother, and told her if anything happened, she would have only herself to blame, and that the act of discharge was solely her own. Notwithstanding this, his mother persisted in her entreaties, and on her signing the form required by Act of Parliament, the prisoner was discharged.

From that time up to the present calamity he had lived with his mother, who had always evinced the greatest possible affection and solicitude for him. He, witness, was in the habit of allowing prisoner a certain sum of money weekly, and for this he came regularly to his house. On Tuesday week he called as usual, when his conduct was so strange that he felt it necessary to send a message to his sister, in reference to him, requesting that she would do something with him. Mr. C——, in explaining what he meant by the "strange manner of the prisoner," stated, that upon leaving his house to go to the butcher's, on the morning in question, he found him standing outside his door like a statue. That he asked him to go into the house, and that when he came back from the butcher's he found prisoner still in the same position. He asked him again to go in, but he said he preferred standing in the sun; and he then went into the yard, where the sun was shining, and remained there for a quarter of an hour or twenty minutes. I then gave him the money I was in the habit of allowing him, and also brought him a coat from the bar which I had promised him. Having a black bag under his arm, I asked him to put the coat into it, but he refused to do so. He kept muttering something to himself; upon which I said—"Young man, I am sorry to see you in this state; you want somebody to control you properly." Upon this he became much excited, said he was an Englishman, that he should fight for his rights, and should allow nobody to control him. His conduct on this occasion was of so extraordinary a character, that I spoke to my wife on the subject, and told her I feared that something dreadful would happen.

It appears that the prisoner was in the habit of carrying a black bag about with him, in which he had a knife and a pistol; that the mother was too fond and indulgent to the prisoner; that he had enlisted twice before he had been first sent to Bethlem, but was discharged as being unfit for the army. He also made his escape from W—— Lunatic Asylum, but was taken an hour afterwards at his mother's house by the keeper, who had come in pursuit of him. The prisoner had exhibited symptoms of insanity before going abroad; one of which was, that he would leave excellent situations without the slightest cause. It further appeared that the prisoner was taken into custody by Inspector D——, of the County Constabulary, for having threatened to shoot a person in the high road, near H——, when the chief constable, before whom he was taken, perceiving him insane, after taking away the pistol with which he was armed, sent him to his mother at L——.

The prisoner was charged with the murder of his mother, and before proceeding to trial the court heard medical evidence upon the point of insanity.

Mr. H——, the surgeon, deposed that the prisoner was received into gaol on the 11th of April last, and that during the time he was there he had frequent opportunities of seeing him; that he was decidedly insane when admitted, and continued so until removed to Newgate; that the prisoner often talked about suicide, and that was his complaint. He said that he had been in two lunatic asylums, and should not be well until he committed suicide.

Mr. J—— C——, the uncle, stated that he considered the prisoner had been a lunatic for the last five years. The state of his mind was first observed after his return from South America. He was confined in Bethlem, and remained there some time, when he was discharged uncured, and went to live at the house of an aunt, where he got worse, and he was removed to the S—— County Lunatic Asylum. From that establishment he was discharged uncured, and went to live with his mother. He was at this time in such a state of mind, and his conduct of such a description, that he, witness, continually urged his mother to have him placed in confinement, for that he was unfit to be at large. He was to have been again confined, and an arrangement was made for that purpose in the evening of the very day on which the dreadful occurrence happened. He was of opinion that the prisoner had not had a single lucid interval since he returned from South America. The prisoner had also been known to stand at the door of the house where he lived, and talk to it for half an hour at a time.

The jury returned a verdict—That the prisoner was of unsound mind, and therefore incompetent to plead.

M—— B——, aged 20 years, was indicted for the wilful murder of E—— B——, her infant child. The prisoner had been married about two years, and was confined of her first child on the day mentioned in the indictment. *The prisoner's husband observed something odd in her manner, and when he went out he begged her mother to take care of her.* The mother went to the prisoner's house, found that she was upstairs, and asked where the child was. The answer from the prisoner, who was upstairs, was, that he was downstairs in the pail. The grandmother went to look, and found the baby stuffed head first in the pail, and quite dead.

Medical witnesses were examined as to the state of the prisoner's mind, and it appeared *that she had been insane before her marriage, and must have been so at the time of her marriage.* Verdict—Not guilty, on the ground of insanity.

A—— M—— was indicted for the murder of E—— A—— M——. It was clearly proved that on the day in question the prisoner had taken her daughter's child, which was two years old, and deliberately drowned it in a well. The defence set up on her behalf was, that at the time she committed the act she was insane.

The surgeon of the gaol proved that on her being brought to prison she was in a very low and dejected state, and extremely feeble, both in body and mind, so that it was not improbable that she might be subject to a fit of temporary insanity. It further appeared that some other members of the prisoner's family had been afflicted with insanity. Further, her conduct immediately after the commission of the act was relied upon as evidence of insanity; it appearing that she had uttered loud cries of lamentation, and exclaimed, "The devil tempted me."—Verdict—Not guilty, upon the ground of insanity.

A—— B——, 37 years of age, was indicted for the wilful murder of C—— G——. When called upon to plead "Guilty" or "Not guilty," he said, "I don't know if I am guilty; I was insane at the time." This was treated as a plea of not guilty, and the prisoner was put upon his trial.

The evidence showed that the prisoner, who lives in a wild district, near L—— B——, had *exhibited such signs of violence, that on that day his wife sent for his father, and begged the old man to sleep with him that night. The father complied. During the night the prisoner was very violent, and early in the morning, after his father had gone to work, he got up, seized a stone-breaker's hammer, and threatened to take the life of an old woman of* 70 *who lived with him and his wife. The deceased escaped from the house, and was followed by the prisoner, still holding the hammer; she escaped into the house of a neighbour, and he, mistaking that into which she had gone, rushed in and inquired for her and his wife, saying he smelt them—that they had ruined his mother, and he would kill them both—that they would cause him to be hung. He then rushed out and went to the house where the deceased was; broke open the door with the hammer, which had been closed against him, and made for the old woman as she was trying to get up the stairs from him. Before she could get quite up, he felled her with a blow of the hammer, and then dealt her two more blows which completely smashed her head, and so extinguished life. Thereupon the prisoner exhibited signs of religious triumph, singing out, "Glory, glory to the Lord! Hallelujah! Hallelujah!"*

The prisoner said nothing in his defence. The governor of the gaol handed in a paper which set forth that *one of his aunts had died insane, and that it was well known at times that he was also insane. The statement as to the aunt was drawn out and made by a surgeon. It was also shown that the prisoner was at times of weak mind, that he often complained of his head, and that sometimes he was very violent.*

The learned Judge placed all these cases before the jury; but after deliberating half an hour, they returned a verdict of—WILFUL MURDER; upon which the prisoner exclaimed, "The Lord's will be done."

The Chief Baron then passed sentence of death in the usual form, and the prisoner was removed from the dock apparently quite insensible to the perilous position in which he stood.

G—— V—— H——, aged 35 years, was indicted for the wilful murder of his wife, J—— H——, by dashing out her brains with a hatchet. The prisoner exhibited great distress of mind during the trial, and was defended by counsel through the humane interference of the Sheriffs. From the statement of the counsel for the prosecution, it appeared that the parties had lived together for many years in great affection, and the only question the jury would have to determine would be, whether at the time the act was committed the prisoner was in such a state of mind as to render him responsible for his actions. He then proceeded briefly to narrate the circumstances under which the crime had been perpetrated, and said that there did not appear to have been the slightest quarrel between the prisoner and his wife; and on the same morning, the prisoner, who, it appeared, had thrown himself into the water, was found in a raving state, and perfectly unconscious of what had occurred. Mr. Baron Martin, before whom this case was tried, said the depositions clearly made out that the prisoner was not in his senses, and it would be only necessary, therefore, to prove the mere facts of the case. The following witnesses were then examined:—

M—— H——, the mother of the prisoner, deposed that she knew the deceased from the time she and the prisoner had been married. On the 17th March, in the evening, they were both at her house, and went away about eleven o'clock;—they were always very affectionate and very friendly together. About six years ago the prisoner was under the care of a physician, on account of pains in his head, and he was like an idiot. For the last three months they had been all terrified by his manner. He used to make grimaces, and looked wild about the eyes. Witness wished him to be put in confinement, but his wife was so fond of him that she objected to part with him. On the morning of the 18th of March last, the prisoner came to her house again; he was wringing wet, as though he had been in the water, and he did not appear to have his senses. Shortly afterwards he was taken into custody. When a boy, he had a severe hurt in his head, and was four times attacked in the manner witness had described.

Mr. D——, the surgeon, deposed that he was called to the prisoner's room about nine o'clock on the morning of the 18th, and he found the body of the deceased lying on the bed in a position as though she was asleep; there was a frightful wound on the head, and the skull was completely battered in on the left side—the blow must have caused immediate death. There was a hatchet in the room, with which, no doubt, from its appearance, the wound had been inflicted. Beside the injury to the head, there was another violent blow on the neck which had dislocated it.

It further appeared, that a short time before the prisoner had been taken by his wife to the surgeon, to whom she stated, in his presence, that she was afraid he would commit suicide, and the prisoner said that he had been tempted to it.

Mr. B——, brother-in-law of the deceased, stated that he met the prisoner on the morning of the fatal occurrence. He was very wet, and he made such grimaces and acted in such a manner that witness hardly knew him. He asked him where he had been, and at first he made no answer, and then began to dance about, and said he had been in the water.

The jury stopped the case. Verdict of Not guilty, upon the ground of insanity.

A—— S—— was indicted for the wilful murder of his mother, J—— S——. It appeared that the prisoner's father and mother lived at a place called C——, and the prisoner resided with them, sleeping in the same bed with his father, his mother occupying a little bed by herself. The prisoner had been reading the "Primitive Methodist's Magazine," and complained of his head. He said studying had made him ill, and he was observed to be strange in his manner. At five o'clock the following morning he got out of bed, and went and looked at his mother, who was lying asleep, and then got into bed again. Shortly afterwards he got up, and obtained a poker and a wooden rolling-pin, and with these commenced beating his mother on the head. His father got out of bed and went to his wife's assistance, when the prisoner turned upon him and beat him upon the head until he became insensible. The prisoner then left the house, dressed only in his shirt, which was bloody, and with the rolling-pin, which was covered with blood, in his hand, went to the house of a neighbour named Wardle, and putting his hand through the window, in which he had the rolling-pin, brandished it about, crying out, "I am the murderer." Wardle, alarmed at his appearance, took his gun down to protect himself, and followed the prisoner until he was taken into custody in a public-house, into which he had gone and seated himself in his shirt, talking wildly, and complaining that his arm hurt him. The deceased was found lying upon her bed in a dying state, and his father was found lying moaning in the bed from the injuries he had received. When the prisoner was taken into custody by the police, and asked what he had been doing, he replied, "Nothing." He was then asked when he had last seen his father and mother, and he replied, "Let me consider; I left them this morning bruised." The deceased died before medical assistance could be procured. A *post-mortem* examination established that she had sustained a compound fracture of both the upper and lower jaws, and she was frightfully beaten on various parts of her body, and had, beyond doubt, died from the injuries she had received. For some time afterwards no rational answer could be obtained from the prisoner, who sang ranters' hymns, and talked wildly.

The surgeon of the gaol, who attended him, expressed his opinion that the prisoner had homicidal mania, and that he was not responsible for his actions.

A broken poker was found near the deceased, which had evidently been broken over her by the prisoner. Verdict—Not guilty, on the ground of insanity.

W—— C—— was indicted for the wilful murder of his mother, A—— C——, at R——. This was a painful inquiry, the prisoner being charged with the death of his own mother, by stabbing her with a large knife in her side, the plea being that at the time of committing the offence the prisoner was labouring under insanity. It appeared from the evidence that some years ago the prisoner was engaged on board a vessel, and whilst so employed he sustained a severe injury of the spine, thus rendering him incapable of working, and causing his mind to be affected. The prisoner occasionally, on any trivial circumstance, became excited, would sometimes strike his mother, but he was not considered to be a dangerous lunatic. The facts of the case were briefly as follows :—The deceased, about noon, left her house to take her husband's dinner, and on her return she found the prisoner and his sister together. The latter remarked to the deceased *that the prisoner's brother had got married at M——. Upon this the prisoner became greatly agitated and enraged, and the deceased used her endeavours to soothe and render him peaceable, but in vain. He seized hold of a dinner-knife which was on the table, and stabbed his mother in her left side; the unfortunate creature sank upon the floor, and was exhausted from the fatal injury she had sustained. Whilst the poor woman was in this pitiable condition, the prisoner exclaimed, "I have done for you, you will not live long!"* He then left the house and went to a neighbour's, being at the time without his hat and coat. He there told the dreadful deed he had committed, and asked the persons in the house to go in and see his mother. Two of the neighbours accordingly ran into the dwelling, followed by the prisoner. The deceased was still upon the floor, when the prisoner remarked, "Aye, she'll soon croak, I've given her plenty!" adding, that he had stabbed her in the back, and she would not be long. The knife lay upon the floor. The neighbours were afraid of meddling with the prisoner, who went out, first putting on his coat and hat. He sat himself down upon the door-step of a neighbouring house, and shortly after a constable came to him; and upon telling him he had to take him into custody for stabbing his mother, the prisoner observed, "Yes, I expected you would be coming," and he went quietly away with the officer, acknowledging what he had done. The deceased lingered for about a week, and died from the wound she had received.

*During the time the surgeon was dressing the wound of the deceased, the prisoner was present; he was much excited, and paced up and down the room, making use of incoherent expressions.* Upon the deceased making a dying statement upon oath in the presence of the prisoner, he again conducted himself in the same strange manner as before. The surgeon's evidence was to the effect that in his opinion the prisoner was decidedly insane. Verdict—Not guilty, on the ground of insanity.

J—— B——, aged 38 years, was indicted for the wilful murder of S—— H——, at C——. The prisoner was a tailor, living with his wife and family at C——, and, until a short time before the commission of the crime with which he was charged, he had always been a peaceable, well-disposed, and respectable man—affectionate to his family, and of a cheerful and happy disposition. *Latterly, however, a change had come over him: he had become much depressed in spirits and reserved in his manners; he no longer took any notice of his children, and said, when he was remonstrated with, that he could not help it. When accosted in the street in a friendly manner by persons considerably his superiors, he made no answer, and on all occasions preserved a sullen silence. This strange behaviour produced at the time the impression that his mind was affected.* The circumstances connected with the commission of the offence were also very strange. The deceased was the second wife of the prisoner's father-in-law, with whom he had always been on very good terms; but about ten o'clock on the morning in question he was seen going into her house, and in a few minutes afterwards she came running out, screaming "Murder." He followed with a poker in his hand, and immediately dealt violent blows upon her head and neck, so that she fell dead. The neighbours came up in a moment, but there was no time to save her, and the prisoner went away. The following evidence shows the state of the prisoner's mind, and that insanity prevailed in his family :—

S—— R—— stated that she lived in the same street as the deceased; that on the 6th of June she saw the prisoner go into the deceased's house, and in two or three minutes the deceased came out, screaming "Murder," followed by the prisoner. She fell down on the side of her face at the bottom of the steps. The prisoner was holding a poker in one hand, and struck her twice with it on the back of the head while she was on the ground. The prisoner's mother was insane, and one of his sisters had been insane.

Mr. J—— S——, a surgeon, was called to support the plea of insanity set up for the defence, and stated that he had known the prisoner's family for many years. His mother was insane. He stated that he could not form any opinion as to the prisoner's insanity; but restlessness at night is a symptom of it, and so, in his opinion, was his conduct at the time when he committed the offence.

Several other witnesses were called for the prisoner, who corroborated the evidence as to his insanity; among them T—— H——, who proved that he had two sisters insane; and Dr. E—— P——, a physician experienced in the treatment of lunatics, who had heard all the evidence in the case, expressed a decided opinion of the prisoner's insanity. Verdict—Not guilty, on the ground of insanity.

H—— S—— was a person belonging to a highly respectable family, aged 26 years, and resided in the house of one R—— M——. During the last two months his manner had been noticed as being very strange, and when spoken to his answers were very incoherent.

From the statement of Mr. R——, the counsel for the prosecution, the prisoner had for some time lodged at the house of the deceased woman. He occupied the back, and she the front parlour. Upon the morning of the 7th of June, at half-past five o'clock, Mrs. A—— L——, a lodger in the house, was alarmed by screams coming from the deceased's room, and she roused another lodger, A—— B——, who immediately rushed downstairs, and there saw the deceased upon her hands and knees upon the third stair, trying to crawl up; she was bleeding from a wound in the throat. The prisoner was standing near her, dressed, and with his hat on; he had an open clasp-knife in his hand, and before B—— could interpose, he laid hold of the woman, and again cut her throat. *Prisoner then coolly closed the knife, and in a wild and insane manner said "the great bear had done it," and that "Mrs. M—— would be a bear," and other incoherent expressions. In the meantime the poor woman was raised up, and placed in Mrs. L——'s room, while B—— went for a policeman and a surgeon; and, upon his return, she was found to be quite dead. Not any motive could be assigned for the commission of the deed.*

Mr. C——, the counsel for the prisoner, stated that he had ample evidence to show the prisoner was a man of unsound mind. He had been a mariner, and received an injury in his head while on ship-board, and from that time down to the present he had been affected in his head. These facts having been deposed to, two medical gentlemen were examined, whose testimony clearly established the prisoner's insanity. Verdict—Not guilty, upon the ground of insanity.

S—— F——, a dressmaker, aged 20 years, a mild-looking and pretty young woman, was indicted for the wilful murder of her illegitimate child, J—— C—— F——, aged 11 months, at S——. This was a most distressing case. The prisoner had put the child out to nurse with a woman eight weeks ago, and was to pay 2*s.* 6*d.* per week for it. She never paid anything for it, and Mrs. L—— told her she would no longer keep the child, and she must get another place for it, which she proceeded to do. She took away the child the same day, saying she was going to take it to C——. The child was very weakly and delicate, and Mrs. L—— thought more than once that it would die, it was so thin and emaciated. The child was not seen again alive; it was found in a pit containing water, with its frock tied over its head, and two bricks pinned in at the back of its head. It further appeared from the evidence of Mr. W——, surgeon, that the child did not die of drowning, but of a blow at the back of the head, either before or at the same time when it was thrown into the pit. The body was not identified, but two witnesses swore positively to the clothes worn by the child. When apprehended, the prisoner denied the crime; but afterwards, when about to be taken before the coroner, she told the constable that she wished him to let her attorney, Mr. E——, know that he need not attend the inquest, as she was guilty of the crime, and it was no use for her to deny it. All the witnesses, except the surgeon, had known the prisoner for several years, and gave her a high character for kindness and humanity; the woman L—— saying, that she was fond of her child—was kind, humane, and respected by every one. It also appeared that the prisoner had been deserted by the father of her child, and that during her pregnancy she suffered from several fits of despondency, and even attempted suicide. It was also proved that insanity prevailed to a considerable extent in her family, her father having died abroad insane, her uncle at the present time confined in a lunatic asylum, and a sister, who is idiotic. Since her desertion by the father of her child, she had formed a connexion with another man, by whom it was alleged she was pregnant, but which, after an examination by a jury of matrons, was proved not to be the case. The defence set up on behalf of the prisoner was, that the identity of the body was not proved; that the child being weakly, had died in her hands from natural causes, from convulsions or otherwise, and that she had thrown the body into the pit to avoid burying it publicly; or that she might have committed the act imputed to her in a fit of insanity, to which malady her family was subject. Several respectable witnesses gave the prisoner an excellent character.

The learned Judge directed the jury that there was not before them the slightest evidence that the prisoner had committed the act imputed to her when in a fit of temporary insanity. His lordship having recapitulated the evidence, and stated its effects to the jury, left them to say whether in their opinion the evidence brought home the capital charge to the prisoner, and he charged them not to flinch from their duty, but, however painful it might be to them, if they believed her guilty they would say so by their verdict. If they had a reasonable doubt upon the matter, they must give the prisoner the benefit of that doubt.

The jury found the prisoner Guilty, but added a strong recommendation to mercy. The Judge then passed the sentence of death in the usual manner, during which he was affected to tears, and promised to forward the recommendation of the jury to the Queen. The prisoner bore the sentence without any visible emotion.

G—— H—— S—— held the office of postmaster at J—— for the last five or six years, and is described as a person of great intellectual attainments, while his hapless wife was highly accomplished, and possessed in her younger days great personal attractions. The accused is 54 years of age, and was placed in the dock before the magistrates at the Guildhall, R——, charged with the wilful murder of his wife by shooting her with a pistol. During the examination he remained perfectly silent, with his face buried in his hands, his elbows resting on the dock, and never once lifting his head to glance round the court. It appeared from the evidence, that on Saturday evening, about six o'clock, the prisoner, accompanied by two females, the one his wife, and the other Mrs. B——, an attendant, drove up to the house of Mr. W—— H—— in a fly, and took apartments, consisting of a parlour and bed-room, for a week. On Tuesday morning, while Mrs. H—— was engaged between seven and eight o'clock in cleaning the hall-door steps, the prisoner came downstairs, and addressing her, said, "Don't drop down dead, Mrs. H——! I have done for my wife; she is an angel!" Mrs. H—— asked him if she was dead; upon which he replied, "No; it would be well for her if she was. Send for a surgeon and the police." He then walked into the parlour, and sat down on the sofa, exclaiming, "She is an angel—she is an angel!" A surgeon was immediately sent for; and as soon as Dr. B—— arrived, Mrs. H—— went to deceased's bed-room with him. They found the deceased lying in the bed, quite quiet, and the clothes were not at all disturbed. She appeared to be asleep. There was no blood at all to be seen on the bed-clothes or pillows. She did not appear to have struggled, or to have been disturbed from her sleep.

C—— H——, a constable, took two pistols from under the mattress upon which the deceased lay. The deceased's wounds were bathed for some time, but she died in about a quarter of an hour. The prisoner appears to have been very kind to his wife during the time they were in the house. No report of firearms, either on the preceding night or on the morning of the deceased's death, was heard by Mrs. H—— or her husband, although they slept in a room over their heads.

Mr. T—— P—— B——, surgeon, stated that about half-past seven on Tuesday morning he was sent for to Mrs. H——'s. Upon proceeding to the deceased's bed-room, he found her lying on her right side in bed, and a wound on the left side of her head, from which both brains and blood had oozed. On cleaning the wound, he found that the skull had been perforated to about the size of his little finger. Having removed two small pieces of bone from the margin of the wound, he cleared it, and endeavoured to arouse the deceased; but she was perfectly insensible. Upon searching the room, he found a brace of pistols between the mattress and the bed. One of the pistols was found to be loaded with two balls, incased in a piece of leather, while the other had been but recently discharged, the hammer being down and the cap split. In a medicine-chest he also discovered a phial containing strychnine, one of the deadliest of poisons. Witness left, and returned again about ten o'clock, when deceased was dead. He subsequently the same morning saw the accused, and conversed with him. He asked witness, "Is she gone?" Witness replied "Yes," and then asked him, "Have you been ill lately, or has there been anything the matter with you?" upon which he said, "It will all come out upon inquiry at H—— P——" (a lunatic asylum in which he had been confined). Witness then asked him when he left that asylum, and he answered, "About ten weeks previously." The conversation then dropped. Witness made a *post-mortem* examination of the deceased, and upon examining the head he discovered that the balls had passed through the middle lobe of the left side of the brain to the anterior lobe on the right side, where their course was stopped by the cranium. The two balls produced he found lodged in the anterior lobe of the brain. Death was caused by concussion and an effusion into the brain, the result of a pistol-shot.

J—— S——, one of the city police, stated that upon asking Mr. H——, in the presence of the accused, if he heard the report of a pistol that morning, or had any idea when the affair took place, upon Mrs. H—— replying in the negative, the prisoner immediately answered, "I have just done it." After being informed by the police that he would be taken into custody for the murder of his wife, he exclaimed, "To murder!" and after a pause said, "There are letters enough to prove all about it."

Mr. W—— H——, the husband of Mrs. H——, in corroborating his wife's evidence added, that about twenty minutes before eight on the Tuesday morning he met the prisoner in the passage, when he asked witness if he had sent for a surgeon and the police. Witness asked him what he had been doing, when he answered, "I have been and murdered my wife; she is an angel!" He then said, "I will go for a doctor and police myself;" but witness replied, "No, sir, you shall not go out of my house," and led him into the parlour. The accused then said, "I shall not be hung for it; it will be worse." Neither that morning nor during the night did he hear any quarrelling between the accused and the deceased. He appeared very much attached to her. He did not hear any report of firearms.

The prisoner was asked by the magistrate if he wished to make any statement; but he still held down his head, as he had done all the time of the inquiry, and only replied by a wave of the hand. He was then committed to take his trial at the M—— Assizes for the wilful murder of his wife.

Superintendent T—— subsequently removed the prisoner to M—— Gaol; and during the time he was in custody, that officer stated that he noticed sufficient to satisfy him that the wretched man was at intervals a decided lunatic. That officer stated that he had known the accused for twenty-five years, from his residing in the immediate locality; and in course of general conversation, he understood, from several rambling statements, that his intention was to have committed self-destruction, when the idea rushed to his mind of murdering his wife. It appears that, from being in affluent, and indeed wealthy circumstances, the poor fellow was very much reduced, and as a last resource procured, through his wife's instrumentality, the postmastership of J——. He frequently betrayed aberration of intellect, and had been out of H——'s A—— only a few months. During the past six weeks, having obtained leave of absence from his duties, he had been travelling about for the benefit of his health. He had, before proceding to R——, resided a week at G——. He was a well educated and talented man, having, before he accepted the office of postmaster, followed the profession of instructing young gentlemen preparatory to their entrance into the universities.

I—— T—— was indicted for the wilful murder of M—— A—— T——, at B——. The following are the facts of the case as they appeared on the trial. The prisoner entered the service of a Mr. T——, who resided at S——, in order principally to wait upon his wife, who was ill, and also to take care of the child in question, which was about 13 months old. The prisoner, however, continued only a short time, and the way she left was as follows:—Something unpleasant having occurred respecting the child's clothes, Mrs. T——, her mistress, remonstrated with her on the subject, but nothing more occurred. However, on the following day the prisoner having been sent on an errand, took the child with her, and from the moment she left up to the time of the murder neither the prisoner nor the child was ever heard of, and at that time it was the 19th of December, when she was traced in company with a man named W——, in a lodging-house at B—— W——; but before intelligence reached the authorities, they had left the town, and nothing more was known of their whereabouts until the beginning of January, when, in a state of misery and destitution, she applied to the relieving officer of B—— A—— for relief, when both she and the child were sent to B—— A—— workhouse. Upon applying to the officer for relief, she said her name was I—— M——, and that she had come from L——, and the father of the child was dead. When taken to the workhouse, the child had a black eye, which the prisoner said was caused by a fall, or by falling from her knee, and that it was only eleven months old. It had no other marks than a black eye, and the prisoner gave various accounts how it was done. She first said she let it fall as she was coming from Darlington; then, that one night when she went out, she gave the child to her husband, and that when she returned and took the child she saw no black eye until the following morning. There were also some marks on the child's back, which she said were done by the father beating it. On the 4th of June, the prisoner went into a room to one of the inmates, and said they must go directly to the child, as it was in a fit. Upon two of the inmates going into the room, the prisoner had the child upon her knee beside the bed. The child made no noise. Mr. H——, the surgeon was sent for, who examined the child. There were no marks of violence on the child then, and it was put to bed with the prisoner. The prisoner had placed the child upon the cold floor; and upon the surgeon telling her it should not be there, she took it up and flung it upon the bed. About eleven o'clock the same night, A—— H——, one of the inmates, went into the prisoner's room; none but the prisoner was there, and she said the child was dying. The surgeon ordered that some persons should sit up with the child all night, but the prisoner said there was no call for that. Upon again inquiring how the child was, the prisoner said, "Nicely." Witness went to breakfast, and upon her return to the room at nine o'clock, she found the prisoner sitting by the bedside with the child upon her knee. Its head was covered with a shawl, which being taken away, the prisoner said the child was dead; the child was taken from her, and upon its being washed, a desperate bruise at the back of the head and a long cut in the forehead were discovered; and on a stone mantel-piece which was in the prisoner's room were marks of blood—and it appeared that the prisoner had dashed the child's head against the mantel-piece, which caused its death. There was blood also on the hearthstone and the boards. Upon being asked how the child's head became bruised, prisoner said it had knocked its head against the bedside—it was an iron bedstead. It also appeared that after the child was dead, the prisoner kept it on her knees as she would a living one. The prisoner said her name was I—— M——, also I—— T——; that the child was her own, but that they called the father M——. Upon a *post-mortem* examination, it appeared there were marks and bruises on the right cheek and body of the child, beside those on its forehead; the side of the head and face were swollen and of a dark livid colour, and there was a fracture near the right side, near the temple. Beneath the scalp was a quantity of extravasated blood, and an extensive fracture on the left side, from the ear to the top of the head. The fracture of the head, together with the extravasated blood, were the cause of death. It would require several blows to cause the fractures, and it was not possible for the child to have done them.

*A brother of the prisoner proved that for several years her conduct and demeanour had been such as to leave no doubt on their minds that she was a girl of weak mind, and approaching almost to idiocy.*

The Judge, in summing up the evidence, commenting as he proceeded, concluded by remarking that the entire transactions in connexion with the prisoner's life and conduct were certainly uncommonly singular for a person of sound intellect. Verdict—Not guilty, on the ground of insanity.

J—— G—— was indicted for the wilful murder of R—— G——, his child, 15 months old. From the evidence, it appeared that the prisoner had lived apart from his wife. She had four children, the youngest of which was the above-named R——. About nine o'clock in the morning, he was seen to go into his wife's house by a woman living next door, who stated that, seeing the prisoner at his wife's house, and hearing a groan, she went in. The prisoner was sitting on a sofa, pulling on his boots, and his wife said, "He has cut my child's throat in bed." Another witness also stated that she heard prisoner's wife say, "He has murdered my child," and who, seeing the prisoner walking quietly down the yard, asked him what he had done, and he replied, "The child is in heaven, and I want to be above as well."

S——, a constable, proved that in taking the prisoner's razor from him, he pointed to one which had blood on it, and said, "*That is the one that did the job, and I believe the Lord ordered me to do it.*" Seven witnesses were examined as to the state of the prisoner's mind, some of whom stated that the disease had manifested itself in other members of his family. To rebut this evidence, five other witnesses were called—viz., the surgeon of the gaol, the governor of the gaol, the police superintendent, the coroner, and another person, all of whom gave evidence in contradiction of the existence of insanity in the prisoner.—Verdict of Not guilty, on the ground of insanity.

Side by side with these distressing cases of fatal suicidal and homicidal mania under the influence of unrecognised and neglected disorder of the psychical functions of the brain, we could place a number of illustrations which have come under our own observation, in which medical treatment *directed to the morbid state of this organ has been followed by the happiest curative results.*

Cases of severe mental despondency and distress—instances of alienation of mind, associated with hallucinations and with apparently chronic and fixed delusions, accompanied by strong suicidal and homicidal feelings—have all yielded to medical treatment, and thus, persons in all grades of life, who, if these conditions had not been fully appreciated, would have fallen victims to their own insane impulses, have been restored to society in a state of mental health. We are fully cognisant of the difficulty of persuading persons inexperienced in these matters, of the importance of attending to the earliest indications of brain and mind disorder. The symptoms which so generally precede the act of suicide—such as depressed spirits, distress of mind, needless alarms and apprehensions as to some foreboding evil, great irritability of temper, and inability to attend to the ordinary occupations of life—excitability, headache, disturbed or sleepless nights, morbidly exaggerated views of the actual ills and circumstances of life—*are, in many cases well-marked signs of acute disorder of the brain, requiring for its treatment the most prompt and energetic treatment.* This type of mental disease is fraught with much more danger to life, and is more frequently associated with the suicidal inclination, than those states of morbid mind connected with paroxysms of mental excitement and maniacal violence.

We must, for the present, remain satisfied with having discharged an important professional duty by directing public attention to this subject. Let our readers look well to the facts previously recorded, and ask themselves the question, whether, if the brain and mental condition of these unhappy suicides had at an early period been recognised and treated, nearly all would not have escaped from a horrible self-sacrifice?

